# Electrospinning Enables Opportunity for Green and Effective Antibacterial Coatings of Medical Devices

**DOI:** 10.3390/jfb16070249

**Published:** 2025-07-06

**Authors:** Saverio Caporalini, Bahareh Azimi, Samir Zergat, Mahdi Ansari Chaharsoughi, Homa Maleki, Giovanna Batoni, Serena Danti

**Affiliations:** 1 Department of Translational Research and New Technologies in Medicine and Surgery, University of Pisa, Via Savi 10, 56123 Pisa, Italy; saverio.caporalini@phd.unipi.it (S.C.); samir.zergat@phd.unipi.it (S.Z.); giovanna.batoni@unipi.it (G.B.); 2 Consorzio Interuniversitario Nazionale per la Scienza e Tecnologia dei Materiali (INSTM), Via Giusti 9, 50122 Florence, Italy; 3 Department of Civil and Industrial Engineering, University of Pisa, Largo Lucio Lazzarino 2, 56126 Pisa, Italy; mahdi.ansari9@ut.ac.ir; 4 Department of Wood and Paper Sciences and Technology, Faculty of Natural Resources, University of Tehran, Daneshkadeh Ave., Karaj 77871-31587, Iran; 5 Faculty of Arts, University of Birjand, End of Shahid Avini Boulevard, Birjand 97174-34765, Iran; hmaleki@birjand.ac.ir

**Keywords:** biopolymer, nanofiber, eco-friendly, natural antimicrobial agents, antimicrobial resistance, sustainable healthcare

## Abstract

The growing antimicrobial resistance and the increasing environmental concerns associated with conventional antibacterial agents have prompted a search for more effective and sustainable alternatives. Biopolymer-based nanofibers are promising candidates to produce environment-friendly antibacterial coatings, owing to their high surface-to-volume ratio, structural adaptability, and tunable porosity. These features make them particularly well-suited for delivering antimicrobial agents in a controlled manner and for physically modifying the surface of medical devices. This review critically explores recent advances in the use of electrospun fibers enhanced with natural antimicrobial agents as eco-friendly surface coatings. The mechanisms of antibacterial action, key factors affecting their efficacy, and comparisons with conventional antibacterial agents are discussed herein. Emphasis is placed on the role of a “green electrospinning” process, which utilizes bio-based materials and nontoxic solvents, to enable coatings able to better combat antibiotic-resistant pathogens. Applications in various clinical settings, including implants, wound dressings, surgical textiles, and urinary devices, are explored. Finally, the environmental benefits and prospects for the scalability and sustainability of green coatings are discussed to underscore their relevance to next-generation, sustainable solutions in healthcare.

## 1. Introduction

Traditional antibacterial agents such as disinfectants and antibiotics have long been fundamental in infection control within healthcare and industrial settings [1,2]. These agents are critical for preventing microbial proliferation and ensuring public health. However, their widespread use has raised significant concerns, particularly regarding microbial resistance [3,4], environmental impact [5,6], and toxicity [7,8]. Microbial resistance is now recognized as a global issue, as bacteria are evolving mechanisms to evade conventional antibacterial strategies, thereby diminishing the effectiveness of many treatments and complicating the management of infectious diseases [9,10]. Additionally, conventional antibacterial agents persist in the environment, accumulating in water bodies and soil, which disrupts ecosystems and contributes to resistance diffusion [7,11]. Furthermore, the toxicity of certain disinfectants and antimicrobial additives—especially with prolonged exposure—poses risks to both human health and the environment [12,13].

Given these challenges, researchers are actively exploring sustainable and biocompatible alternatives [13,14]. Polymer-based electrospun fiber coatings represent a promising solution [15]. These materials offer a combination of high surface area, tunable porosity, and mechanical robustness, which makes them ideal platforms for incorporating antimicrobial agents [16,17]. Unlike traditional approaches, these fibers can facilitate controlled drug release, enhanced interaction with microbial cells, and prolonged antibacterial efficacy.

Electrospinning (ES) is a versatile technique for the formation of nano/ultrafine fibers that facilitates the incorporation of bio-based polymers, natural antimicrobial additives (such as essential oils and plant extracts), and environmentally friendly solvents. When incorporated into medical devices such as urinary catheters, wound dressings, or surgical drapes, these coatings significantly reduce the risk of biofilm formation and device-associated infections, such as catheter-associated urinary tract infections (CAUTI) [18,19,20]. Moreover, by replacing chemical-laden disinfectants, these fibers support environmental sustainability and reduce chemical waste [21]. Fiber coatings exhibit superior performance compared to conventional antibacterial agents. Unlike antibiotics, which chemically target specific bacterial mechanisms and contribute to resistance development, electrospun fibers employ multiple antibacterial strategies, such as sustained drug release and enhanced physical interactions with bacterial cells [22,23,24,25]. This multifaceted approach reduces the likelihood of resistance emergence. Additionally, their structural flexibility allows for customized coatings tailored to specific applications, including wound dressings, filtration systems, and implant coatings. Polymer-based fibers also offer sustainability advantages. Unlike conventional antibacterial agents that usually rely on non-renewable resources as well as polluting and energy-intensive manufacturing [26,27], electrospun fibers can be synthesized from biodegradable and renewable polymers, minimizing environmental impact [28]. Their controlled-release mechanisms ensure long-term efficacy, reducing the need for frequent reapplications and lowering overall costs in healthcare and industrial applications. However, despite their potential, challenges persist, particularly regarding the long-term efficacy of coatings and the possibility of including microbial resistance [24]. Moreover, polymer-based electrospun fibers face challenges related to large-scale production, regulatory approval, and cost-effectiveness. While initial production costs may be higher than conventional agents, their durability and reduced application frequency can offer long-term economic benefits. Addressing these challenges through research and technological advancements will be critical in realizing the full potential of polymer-based fibers’ antibacterial coatings [29]. Consequently, ongoing research into green nanotechnologies, biodegradable materials, and scalable fabrication methods is crucial for overcoming these limitations and fully harnessing the potential of polymer-based fibers [25,26,27].

This review provides a comprehensive evaluation of polymer-based electrospun fibers as green alternatives for antibacterial coatings, addressing the critical limitations of conventional agents. By synthesizing recent advancements in material science, sustainability, and antimicrobial technology, it highlights how these innovative fibers can effectively combat infections while minimizing environmental and health risks. As the demand for safer and more sustainable antimicrobial strategies grows, this review offers valuable insights to guide future research, development, and implementation across biomedical and industrial domains.

## 2. Fundamentals of Polymer-Based Electrospun Fibers

### 2.1. Definition and Characteristics of Electrospun Fibers

Recent advancements in nanotechnology have significantly increased the development and biomedical application of nanoscale materials, with polymer-based nanofibers emerging as particularly promising candidates [30,31]. Fibers are defined as one-dimensional, elongated structures. Electrospun fiber diameters are known to range from approximately 10 nanometers to a few micrometers [32]. Properly, nanofibers are defined with diameters lower than 100 nanometers, whereas the electrospun fibers usually obtained are so-called “ultrafine” by showing diameters between 100 nanometers and a few microns. Their unique physicochemical characteristics, such as a high surface area-to-volume ratio, remarkably interconnected porosity, and advanced mass transport properties, make electrospun fibers highly suitable for various biomedical fields [33,34]. These structural features enable nano-to-ultrafine fibers to fulfill essential roles in tissue engineering [35], drug delivery [34], and wound dressing [36]. They can function both as scaffolds and as delivery platforms for bioactive agents, including antibiotics, peptides, or growth factors, thus enabling localized and controlled therapeutic delivery [37]. Notably, by having diameters in the range of collagens’ ones, their ability to mimic the fibrous architecture of the extracellular matrix promotes cell adhesion, proliferation, and tissue regeneration, thereby further enhancing their biomedical applications.

### 2.2. Methods of Nano/Ultrafine Fiber Fabrication

**Figure 1:**
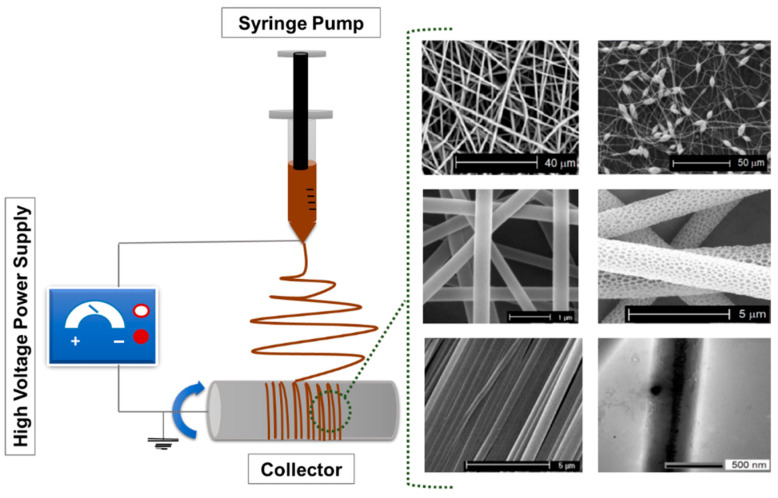
Schematic representation of a typical ES setup composed of a high-voltage power supply, a syringe pump, a spinneret (needle), and a grounded collector. The figure includes representative scanning electron microscopy (SEM) micrographs of diverse electrospun fibers reproduced. Adapted from Ref. [39].

The fabrication of polymer-based ultrafine fibers involves various advanced techniques, each with distinct advantages and limitations tailored to specific applications. Common methods include ES, rotary jet spinning, and solution blow spinning. These approaches differ in how they generate fiber structures and influence specific properties, such as fiber diameter, porosity, surface area, and mechanical integrity, thus making them suitable for diverse biomedical, environmental, and industrial applications. Among these methods, ES is the most widely used technique due to its simplicity, versatility, and precision. The typical setup includes a syringe pump, a metal needle, a high-voltage power supply, and a collector [38]. Key process parameters, such as polymer solution properties, process conditions (including voltage, flow rate, and needle-to-collector distance), as well as ambient factors like temperature and humidity, affect the morphology and functional properties of the resulting fibers. Figure 1 shows a schematic representation of a typical ES setup.

Beyond ES, other methods are gaining interest for their scalability and lower environmental impact. Rotary jet spinning (also known as centrifugal spinning) uses centrifugal force instead of electric fields, providing a solvent-compatible, high-throughput, and voltage-free alternative. Solution blow spinning, which utilizes compressed gas to stretch polymer solutions into fibers, allows for rapid production with potential applications in filtration media and disposable medical textiles. As sustainability becomes a key consideration, these fabrication methods are being optimized to reduce environmental impact. Traditional ES systems often rely on volatile organic solvents, which raise concerns about emissions and resource waste. However, modern eco-friendly ES systems, including closed-loop solvent recovery and capture and reuse of solvents, are taken into consideration as they may considerably reduce emissions and operational costs.

Additionally, the use of water-based or bio-derived solvents further enhances the safety and environmental compatibility of electrospun fibers [40,41,42]. Innovation in fiber fabrication continues to evolve within a very dynamic scenario. Innovations such as coaxial ES for core–shell fiber structures, multi-fluid spinning, and the creation of functionalized ultrafine fibers are driving breakthroughs in some specific applications, such as drug delivery and tissue engineering [43]. The integration of these advanced manufacturing techniques with sustainable practices ensures that the development of electrospun fiber remains both high-performing and environmentally responsible [44,45]. A deep understanding of the diverse fabrication techniques, along with their environmental implications, is vital to engineer advanced fiber-based materials that are not only high-performing and customizable but also scalable and eco-friendly. Therefore, convergence of technological and green innovation can support the growing demand for scalable, safe, and effective antibacterial coating solutions.

### 2.3. Types of Polymers

Polymer-based ultrafine fibers provide a versatile platform for antibacterial coatings due to their high surface area, tunable porosity, and excellent biocompatibility. These characteristics make them ideal for delivering antimicrobial agents to targeted surfaces in a controlled manner [46]. In the context of wound dressings, for instance, these fiber coatings have the potential to facilitate sustained release of antimicrobial agents directly at the wound site, thereby enhancing infection control and accelerating healing processes [47]. Similarly, fiber coating on medical implants has been shown to inhibit bacterial colonization and biofilm formation, thereby reducing the risk of post-surgical infection and enhancing implant longevity and performance.

A wide range of polymers can be used in fiber production, each one offering distinct advantages depending on the intended application [48]. Synthetic polymers, including polyethylene (PE), polycaprolactone (PCL), polyethylene oxide (PEO), and polyvinyl alcohol (PVA), offer predictable and customizable properties [49,50]. PE is a petrol-based polymer known for its chemical inertness, flexibility, and durability, making it suitable for packaging, medical devices, and implants [51]. PCL is widely used in drug delivery and tissue engineering due to its biocompatibility and slow degradation profile [52,53]. PEO is favored in hydrophilic applications like wound dressings for its non-toxic, water-soluble nature [54]. With its remarkable chemical resistance, biocompatibility, and capacity to form hydrogels, PVA is used in both tissue scaffolds and wound care systems [55].

In contrast, biobased polymers, derived from renewable resources, are gaining prominence due to their possible biodegradability and exceptional biocompatibility with living tissues [56]. Examples include polylactic acid (PLA) [57], polyhydroxyalkanoates (PHA) [36], and cellulose [58], which are among the most studied bio-derived polymers for biomedical use. These polymers are biodegradable, ensuring their environmental sustainability, and are often produced via sustainable methods such as microbial fermentation [39,59]. Their mechanical strength, chemical stability, and healing-promoting properties make them especially attractive for applications like wound healing and tissue regeneration. With their biodegradability and inherent compatibility with living systems, biobased polymers represent a significant transition toward more sustainable and effective materials in healthcare and other fields [60,61,62]. Through the judicious selection of the polymer matrix, researchers can optimize the performance and functionality of fiber coatings for antibacterial applications. One strategy involves the integration of synthetic polymers, renowned for their mechanical strength, with natural polymers, which possess inherent biological activity. This integration results in the formation of hybrid fibers that exhibit a combination of durability and bioactivity. Such a methodology facilitates the development of advanced materials that can effectively inhibit bacterial growth while promoting tissue regeneration and reducing environmental impact. The incorporation of biopolymers in the manufacturing of fibers enhances the biocompatibility and sustainability of coatings, aligning with the principles of green chemistry and sustainable development goals [28].

## 3. Green Approaches in the Synthesis of Polymer-Based Electrospun Fibers

### 3.1. Sustainable Polymer Sources

The ES process frequently employs synthetic polymers, such as polyurethane (PU), PVA, and PCL. Although these materials are extensively used, they typically exhibit a deficiency in bioactivity, which constrains their interactions with biological systems [63,64]. In response, recent research has focused on identifying sustainable polymeric sources that not only address ecological concerns but also enhance the biological performance of ultrafine fibers. Sustainable polymers are typically derived from renewable resources such as plants, biomass, and industrial or agricultural waste. These biodegradable and bio-based materials provide a more sustainable alternative to petroleum-derived polymers and are consistent with global initiatives aimed at reducing environmental footprints and promoting circular bio-economy [65,66]. The integration of sustainable polymers into fiber fabrication enhances both the ecological profile and the bioactivity of ultrafine fibers. It also improves their functionality in applications such as antimicrobial coatings, wound healing, and regenerative medicine. This approach supports global sustainability and public health initiatives while addressing critical challenges in healthcare, agriculture, and environmental protection. Furthermore, it promotes the development of ethical, resilient, and low-impact material technologies [67,68,69,70].

Cellulose, the most abundant natural polymer, is one of the most promising candidates for sustainable fiber production [71]. Sourced from wood pulp, cotton, or bamboo, cellulose is biodegradable, renewable, and highly biocompatible, making it well-suited for biomedical applications such as wound dressings, implants, and food packaging [72,73]. Cellulose hydroxyl-rich backbone provides numerous reactive sites, enabling the synthesis of a wide variety of functional derivatives tailored to specific applications. Its excellent mechanical properties and inherent compatibility with biological systems make cellulose-based ultrafine fibers especially promising for antimicrobial coatings and other healthcare uses [74,75]. ES and solution-blowing have been successfully employed to produce cellulose ultrafine fibers, for which they can be considered greener manufacturing technologies than conventional manufacturing processes. These methods offer significant advantages, including reduced energy consumption and waste generation [37,76]. The resulting fibers demonstrate a high surface area and porosity, characteristics that significantly enhance their efficacy in biomedical applications, including wound healing and tissue regeneration [77]. Moreover, ultrafine cellulose fibers showed remarkable antimicrobial activity when functionalized with metal nanoparticles or bioactive agents. This functionalization significantly expands their potential in advanced antibacterial coatings and sustainable healthcare materials [78].

Chitosan (CS), another key biopolymer, is obtained from the deacetylation of chitin found in crustacean shells or fungi. Known for its intrinsic antimicrobial properties and cationic nature in acidic conditions, CS can disrupt bacterial cell membranes, making it effective against a broad spectrum of pathogens [79]. Electrospun CS-based fibers exhibit excellent antibacterial activity, biocompatibility, and biodegradability, making them particularly attractive for wound healing, tissue engineering, and drug delivery systems. Its renewable origin further strengthens CS’s appeal in sustainable material development [60]. PHAs are a class of biodegradable polyesters produced via microbial fermentation of renewable carbon sources. PHAs are versatile, mechanically robust, and can be tailored by controlling their monomer composition [80,81]. 

Notably, bioactive PHAs with intrinsic antibacterial functionality can be synthesized by incorporating functional groups such as thioesters during fermentation. For example, PHA-thioesters synthesized using Pseudomonas putida KT2442 and fatty/thiol acid substrates have demonstrated both antibacterial activity and resistance to microbial degradation. These characteristics make thiolated PHAs highly promising for advanced antimicrobial fiber applications [59,82,83]. In addition to cellulose, CS, and PHAs, other biodegradable and bio-based polymers, including PLA, starch-based polymers, and lignin-derived polymers, are increasingly being explored for applications in antimicrobial fibers. PLA, derived from fermented plant sugars, offers excellent processability and biodegradability. Starch-based polymers are cost-effective, readily available, and degrade rapidly in natural environments. Lignin, a by-product of the paper industry, possesses intrinsic antioxidant and antimicrobial properties, making it an attractive additive in fiber formulations [84,85]. Protein-based polymers, such as those derived from soy or silk, provide additional renewable options with unique mechanical and biological features [86,87]. Recent advancements in green biomaterials have spotlighted plant proteins as promising candidates for sustainable electrospun fibers in biomedical applications [88]. Plant-derived proteins such as zein, soy protein isolate, and wheat gluten offer tunable mechanical and biochemical properties, high biodegradability, and inherent biocompatibility. In particular, electrospun nanofibers based on plant proteins have demonstrated excellent potential for wound healing, drug delivery, and tissue engineering [89]. These fibers can be functionalized or blended with synthetic or other natural polymers to enhance processability and tailor performance characteristics. As reviewed by Kaluata et al. [90], such systems exemplify the convergence of sustainability and functionality, making them highly relevant for next-generation biomedical coatings and scaffolds. The transition toward sustainable polymer sources in fiber synthesis is essential for reducing the environmental impact of material production and promoting the development of eco-friendly, high-performance fibers. By utilizing renewable, biodegradable, and inherently bioactive polymers, researchers are able to design next-generation antimicrobial coatings that effectively address both health and environmental challenges. This transition toward green materials supports the broader vision of sustainable innovation in fiber technologies, particularly in the context of medical and healthcare applications.

### 3.2. Green Solvents

A fundamental component of green manufacturing is the use of environmentally friendly solvents and processing methods that minimize harm to human health and ecosystems. Traditional solvent-based ES methods frequently depend on volatile organic compounds (VOCs) and hazardous chemicals, including dimethylformamide (DMF) and chloroform, which present considerable risks to human safety and the environment [91,92,93]. In contrast, green ES methodologies prioritize the utilization of environmentally benign solvents, such as water and bio-based solvents derived from renewable resources. These solvents are generally characterized by low toxicity and demonstrate minimal environmental impact, thereby aligning with the principles of sustainable production [89,90,91,92]. ES, a versatile and scalable technique for nano/ultrafine fiber fabrication, can be adapted to more sustainable approaches such as using water-based solutions or solvent-free melt ES, both of which reduce reliance on organic solvents and mitigate environmental pollution [94]. Water-based ES has gained considerable attention as an environmentally friendly alternative, particularly in response to growing environmental concerns and the necessity to enhance workplace safety.

Water-based ES has demonstrated effectiveness in processing hydrophilic polymers such as PEO, PVA, alginate, collagen, CS, and cellulose derivatives [95]. However, the scope of this method is limited by the range of polymers that are soluble in water, which constrains its broader adoption. The choice of an appropriate solvent is a pivotal factor in the ES process, as many polymers require highly specific solvent systems for proper dissolution and fiber formation. Unfortunately, solvents with strong solvation properties are often toxic, non-recyclable, and environmentally hazardous [92,93].

To address these issues, scientists have investigated greener alternatives such as U.S. FDA Class 3 solvents, including acetic acid and (bio)ethanol. These solvents have been used with biopolymers like gelatin and CS, demonstrating lower global warming potential and improved environmental compatibility [93]. Binary solvent systems have emerged as effective approaches for optimizing fiber quality while ensuring environmental safety. A notable example is an acetic acid/water mixture used for cellulose acetate. In other cases, a transition to less harmful solvent mixtures has been attempted, e.g., using dimethyl sulfoxide (DMSO)/acetone blends to dissolve and electrospin polyvinylidene fluoride (PVDF), a non-biodegradable synthetic polymer, in place of the most toxic and expensive hexafluoro isopropanol (HFIP). These systems facilitate the production of uniform fibers while simultaneously mitigating solvent toxicity [92].

In recent years, a growing part of the electrospinning research has addressed the environmental concerns of ES solvents, aiming at replacing toxic solvents with greener alternatives. Recent advances in green chemistry have led to the exploration of several innovative solvents for ES, particularly targeting sustainability and biopolymer compatibility. In the case of cellulose, typical solvents are N-Methylmorpholine N-oxide (NMMO) and Dimethylacetamide/Lithium Chloride, since cellulose dissolution in conventional solvents is impeded by the strong inter- and intra-molecular hydrogen bonds. As alternatives, ionic liquids (ILs), such as 1-butyl-3-methylimidazolium acetate (BmimAc), have garnered attention for their ability to dissolve cellulose without derivatization and low volatility, though challenges in their viscosity, as well as recovery and recycling, remain [96]. In addition, most ILs still pose issues related to aquatic toxicity, poor environmental biodegradability, and cytotoxicity.

**Figure 2:**
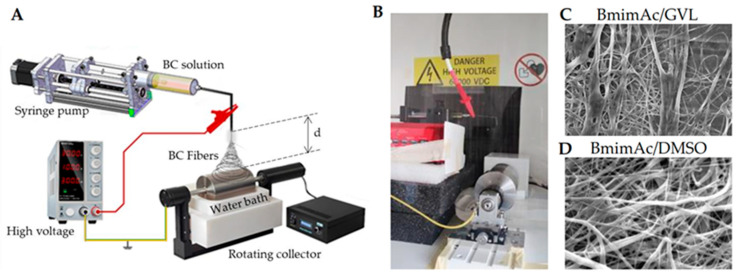
Bacterial cellulose (BC) ES in IL: (**A**) schematic the ES setup including a coagulation bath to remove IL; (**B**) photograph of the setup; (**C,D**) representative scanning electron microscopy (SEM) micrographs of BC fibers obtained using either (**C**) DMSO or (**D**) GVL as a co-solvent of BmimAc. Adapted from Ref. [100].

Deep eutectic solvents (DESs) have attracted growing interest as innovative and green alternatives to conventional volatile solvents. By combining two or more liquid or solid components, DESs result in a liquid characterized by low volatility, low toxicity, and biodegradability, with its constituents derived from natural compounds [97,98]. Especially those based on choline chloride with hydrogen bond donors like urea or lactic acid, offer low toxicity, biodegradability, and tunable properties, making them promising for bio-based fiber production. These unique features make DESs especially attractive for green and sustainable ES processes. Although research in this area is in its early stages, recent studies have demonstrated the feasibility of using DESs in conjunction with biopolymers. For instance, Sousa et al. demonstrated the successful ES of agar using a DES composed of choline chloride and urea. This formulation improved the viscoelastic properties and electrospinnability of an agar solution. Moreover, the addition of PVA further enhanced fiber formation, highlighting DESs as valuable tools for expanding the scope of green ES applications [99]. Gamma-valerolactone (GVL), a biomass-derived solvent with excellent solvency and low volatility, stands out for its ability to process lignocellulosic materials and support fiber formation when combined with co-solvents [100]. GVL was successfully used by Vasili et al. to replace DMSO as a co-solvent of BmimAc to electrospun bacterial cellulose (BC) fibers (Figure 2).

One weakness of low volatility solvents in ES relies on the need to use an antisolvent to remove them from the fibers, usually water. This is performed by using a water bath where the fibers are collected. Therefore, the water containing ILs or DES must be treated to remove and possibly recycle the expensive and sometimes toxic ILs. DESs can enable a different approach, depending on the components, e.g., those with urea and choline chloride, as the wastewaters could potentially allow direct use in agriculture.

Additionally, supercritical CO_2_ and switchable solvents are emerging as innovative platforms for controlled fiber morphology and solvent reuse. Together, these next-generation solvents represent a shift toward more sustainable and efficient ES processes for biopolymer-based textiles and materials [101].

### 3.3. Green Additives and Functionalization Techniques

The incorporation of environment-friendly additives and functionalization techniques is essential to enhance the antibacterial properties of fibers while maintaining sustainable manufacturing practices. Natural antimicrobial agents, such as plant extracts and essential oils, present sustainable alternatives to synthetic chemicals. These agents demonstrate significant antibacterial activity with minimal ecological impact [94,102]. These bioactive compounds can be incorporated into fiber formulations using eco-friendly processing methods that preserve their biological efficacy and ensure safety and biocompatibility [103]. Functionalization techniques, such as surface coating, grafting, and chemical immobilization, enable controlled and targeted release of antimicrobial agents. This approach optimizes therapeutic efficiency while minimizing off-target effects and environmental exposure [44]. By leveraging green additives and functionalization strategies, researchers can engineer ultrafine fibers with tailored antimicrobial properties, addressing the increasing demand for sustainable solutions across sectors such as healthcare, environmental remediation, and consumer products.

In biomedical applications, bioactive nanoparticles have garnered significant attention due to their broad-spectrum antimicrobial effects. Among them, silver (Ag), copper (Cu), and zinc (Zn) nanoparticles have been widely explored for their ability to release ions that disrupt bacterial cell membranes, impair metabolic activity, and inhibit replication. For instance, silver nanoparticles (Ag-NPs) are recognized for their ability to disrupt vital cellular processes in bacterial organisms, whereas Cu and Zn ions demonstrate comparable mechanisms by destabilizing cell walls and influencing enzymatic pathways [18]. Bioactive glasses, also known as glass ceramics, are another green additive with growing interest in biomedical engineering. These materials form biological bonds with bone tissue and are widely used in orthopedics and dentistry due to their compatibility with living tissues [104]. Additionally, composite membranes such as CS/polyethylene glycol (PEG) enriched with silver nanoparticles demonstrated strong antibacterial activity while remaining biocompatible [104,105].

Electrospun membranes incorporating antibacterial agents have shown great potential in wound care applications. For example, PVA fiber meshes loaded with Ag-NPs provide effective antibacterial action, while PLLA and PLLA/PEG membranes coated with CS have demonstrated enhanced hemostatic properties and improved antimicrobial performance [104,105]. Natural antimicrobial compounds are increasingly being considered as replacements for conventional antimicrobial coatings. These agents can be integrated into ultrafine fiber meshes to exhibit intrinsic antimicrobial properties, thereby inhibiting bacterial proliferation and the formation of biofilms. Chitin and its derivative CS are among the most extensively studied, exhibiting bactericidal effects through interactions with microbial membranes. In addition, cationic polymers, derived from natural sources, further enhance the antimicrobial efficacy of ultrafine fibers by interacting with the bacterial cell membrane, disrupting its integrity, and thereby inhibiting bacterial growth [106]. Embedding these bioactive molecules into fibers enables sustained and modulated release, which is vital for prolonged antimicrobial action, particularly in medical devices, wound dressings, and tissue engineering scaffolds. By integrating natural agents with advanced ultrafine fiber technology, it is possible to design multifunctional surfaces that inhibit microbial adhesion through physical, chemical, and electrostatic means, while simultaneously promoting cell adhesion and proliferation [22,107].

## 4. Antibacterial Properties of Polymer-Based Electrospun Fiber

### 4.1. Mechanisms of Antibacterial Action by Ultrafine Fiber

Polymer-based electrospun fibers can exhibit antibacterial activity through multiple mechanisms, including physical disruption, chemical interactions, electrostatic forces, controlled release of antimicrobial agents, and inhibition of biofilm formation. Ultrafine fibers can physically damage bacterial cells by penetrating or disrupting their membranes. The nanoscale dimensions, high surface area, and sharp morphology of fibers contribute to this mechanical action, particularly effective against Gram-negative bacteria with thinner, although more complex, cell walls. Parameters, such as fiber diameter, surface roughness, alignment, and density, can significantly influence antibacterial efficacy; specifically, smaller diameters and denser networks enhance bacterial contact and facilitate membrane rupture [60,108,109,110].

Certain polymers exhibit intrinsic antibacterial properties. For instance, CS disrupts bacterial cell walls through interactions with negatively charged components of the membrane, resulting in membrane disruption, bacterial death, and cell lysis [111,112]. Additionally, embedding Ag-NPs into fibers allows controlled release of silver ions, which interfere with bacterial DNA replication and protein synthesis [113,114,115,116]. Antimicrobial peptides (AMPs) can also be incorporated into fibers to disrupt membranes and inhibit enzymatic functions [47,117], offering broad-spectrum antibacterial activity and reducing the risk of resistance development [118]. Positively charged fibers interact with negatively charged bacterial membranes, causing membrane destabilization and cell death [119,120]. This can be enhanced by incorporating quaternary ammonium compounds or by applying surface treatments like plasma modification to adjust surface charge and improve selectivity and antibacterial efficiency [121,122]. Electrospun fibers can also serve as carriers for the gradual release of antibacterial agents, such as Ag-NPs, AMPs, or metal oxides. This sustained release enables long-term protection against microbial colonization and infection, particularly in wound healing or implant applications [114,116,120,121,122,123,124,125,126].

Finally, electrospun fibers have the potential to inhibit the initial adhesion of bacteria and disrupt mature biofilms, which are dense bacterial communities intrinsically resistant to antibiotics. CS-based ultrafine fibers and Ag-NP-loaded fibers have demonstrated strong anti-biofilm activity by releasing ions or interacting with the biofilm matrix [127,128,129,130]. Coatings of medical devices using such fibers have effectively reduced infection rates associated with biofilm formation [131,132]. By integrating these synergistic mechanisms, polymer-based ultrafine fibers offer a versatile and effective platform for antibacterial applications in medical devices, wound care, and tissue engineering.

### 4.2. Factors Influencing Antibacterial Activity

The antibacterial performance of polymer-based fibers is influenced by multiple factors, including polymer composition, fiber morphology, surface modification, release kinetics of active agents, environmental conditions, bacterial strain, and synergistic effects with other antimicrobial agents [133]. Polymer composition is fundamental to antibacterial efficacy. Certain polymers, such as CS and its derivatives, possess intrinsic antimicrobial properties and can act directly on bacterial cell walls or interfere with microbial metabolism without requiring additional agents [134]. In contrast, commonly used polymers like PVA and PCL offer good mechanical properties, but they lack inherent antibacterial activity [135,136]. Fiber morphology, particularly diameter, surface area, and porosity, also plays a critical role. Ultrafine fibers with smaller diameters exhibit higher surface area-to-volume ratios, enabling closer contact with bacterial cells and enhancing antimicrobial action [19,137]. Moreover, porous structures and rough surfaces increase adhesion sites for bacteria, improving antibacterial efficiency [119].

Surface modification is a powerful strategy to tailor antibacterial performance. Techniques like functionalization, coating, chemical grafting, and plasma treatment allow for improved bacterial interaction or resistance, often through the immobilization of antimicrobial agents that disrupt bacterial membranes or inhibit metabolism [53,127,138,139]. Additionally, such modifications can enable sustained release of active agents and enhance stability under harsh conditions. Release kinetics are equally critical. Controlled-release systems support sustained antibacterial effects and diminish the risk of resistance associated with burst-dose exposures [140]. Optimizing release profiles through polymer matrix selection, carrier systems, or environmental triggers (e.g., pH, temperature) is essential for applications such as wound dressings or implants [111,140]. Environmental conditions, including pH, temperature, and humidity, significantly affect both fiber stability and the activity of loaded antimicrobial agents. These factors influence degradation rates and release profiles, as well as bacterial adhesion and viability [23,141,142]. Environmentally responsive coatings incorporating smart polymers or phase change materials can adapt to external stimuli, maintaining antibacterial efficacy under dynamic conditions [111,143,144].

Bacterial strain and concentration also impact fiber efficacy. Differences in cell wall structure (e.g., Gram-positive versus Gram-negative) and metabolic pathways lead to varied susceptibility [111,119]. At high bacterial loads, the implementation of more potent or sustained-release systems may be necessary to preserve therapeutic efficacy, particularly in clinical settings [137]. Finally, synergistic effects with non-antibiotic agents represent a promising route for enhancing antibacterial efficiency. Incorporating Ag-NPs, zinc oxide (ZnO-NPs), bioactive glass, antimicrobial peptides, or natural compounds like essential oils can boost activity through mechanisms such as membrane disruption, oxidative stress induction, or metabolic interference, often without promoting resistance [145,146,147,148,149]. For example, PCL ultrafine fibers functionalized with Ag-NPs or bioactive glass have demonstrated success in preventing post-surgical infections and promoting tissue regeneration [23,150].

### 4.3. Biodegradation and Environmental Impact

In the development of environmentally sustainable fiber-based systems, biodegradability and environmental impact are crucial factors to consider. Achieving an equilibrium between antimicrobial efficacy and ecological compatibility is essential to reduce the risk of contamination and support ecosystem health. The design of fibers with customized degradation kinetics facilitates their application in diverse contexts, from short-term biomedical use to long-term agricultural applications. Biodegradability refers to the breakdown of fibers into non-toxic compounds through microbial, enzymatic, or hydrolytic processes, significantly reducing waste and pollution, particularly in disposable biomedical applications such as wound dressings [151,152]. Degradation profiles can be tuned for specific needs. Rapid degradation is suitable for transient medical applications, while materials requiring prolonged durability, such as agricultural mulching films, benefit from slower degradation profiles [119,153]. To ensure environmental safety, it is crucial to evaluate degradation by-products and their potential effects on soil, water, and air. In this regard, life cycle assessment (LCA) provides a comprehensive understanding of the environmental footprint, covering the full product, from raw material sourcing to disposal. LCA assesses key indicators such as energy consumption, greenhouse gas (GHG) emissions, and resource use, guiding the development of low-impact materials [151,154,155].

For instance, the total energy required to produce 1 mL of electrospun polymer solution is estimated to be approximately 9.14 × 10^5^ J (254 W·h), accounting for the energy used by the syringe pump, high-voltage power supply, and solution preparation. In contrast, pressurized gyration, a more efficient and scalable fiber production method, consumes only 5.19 × 10^5^ J (144 W·h) per ml and is capable of mass production using water-based polymer solutions, further reducing the environmental footprint. In terms of emissions, substituting traditional polymers with PLA has been shown to reduce GHG emissions by up to 67% during manufacture compared to petroleum-based plastics. Additionally, phase separation methods that incorporate supercritical CO_2_ drying (instead of freeze-drying) achieve a 50% reduction in environmental impact, though they may demand high energy input (up to 1.43 × 10^9^ J per ml in some configurations) [156]. High voltage is crucial for fiber jet formation, but its energy consumption is mostly negligible (<0.1 Wh, using 25 kV for 4 h), due to the low current used in electrospinning (order of nA-µA). This is because the polymer solution generally is not a good conductor, and the electrospinning setup has very high electrical resistance due to air gaps, dielectric properties of solvents, and small needle diameter. The real energy burden in ES pertains to ancillary equipment like pumps, collectors, heaters, or climate control, not the high-voltage supply itself. The highest contributors to energy consumption in ES are solvent production and recovery (e.g., ILs like BmimAc, co-solvents like DMSO or GVL), solvent losses (to air or wastewater treatment), biomass extraction/purification (especially for BC or mycelium), post-processing energy use (e.g., coagulation, drying, annealing), and collector systems (e.g., motors, heating, or vacuum). Energy consumption becomes relevant in multi-jet industrial systems or needleless electrospinning, like wire or bubble-based, due to the large setups and the necessity of climate control.

Biopolymer-based ultrafine fiber coatings incorporating bioactive nanoparticles represent a sustainable alternative in healthcare, delivering effective antibacterial performance while minimizing environmental burden. This approach demonstrates the potential for cost reduction and the improvement of public health outcomes [157,158]. Notable examples include ultrafine fibers derived from cellulose, CS, and PHA, biopolymers known for their biodegradability and biocompatibility. Moreover, incorporating eco-friendly design principles into fiber production processes, such as using renewable resources, reducing energy input, and minimizing waste, further reduces environmental impact. These practices align with global sustainability goals and advance the responsible development of fiber-based technologies [14,159].

## 5. Biomedical Applications of Antibacterial Fiber Coatings

### 5.1. Fibrous-Based Coating for Wound Dressings

Antibacterial coatings on wound dressings function as a specialized interface between the external environment and the wound bed. These coatings fulfill a dual function: they provide a barrier against microbial invasion while facilitating the sustained release of antimicrobial agents directly at the site of injury. By doing so, they modulate the local microenvironment, enhance infection control, and promote tissue regeneration throughout the wound healing process. Electrospun fiber membranes have emerged as a promising platform for advanced wound dressings due to their high porosity, large surface area, and structural similarity to the extracellular matrix (ECM) (Table 1). For instance, collagen-coated electrospun PCL ultrafine fibers functionalized with Ag-NPs have demonstrated robust antimicrobial efficacy and enhanced biocompatibility. These features position them as viable coatings for conventional substrates, such as cotton gauze and other medical textiles, thereby enhancing wound care outcomes significantly [160]. Similarly, electrospun silk fibroin (SF) membranes coated with polydopamine (PDA) have shown markedly improved wound healing in vivo, substantiating their application as bioactive coatings for standard dressing materials [161]. These findings highlight the effectiveness of depositing bioactive fiber layers onto commercial wound dressings, thereby combining structural support with advanced therapeutic functionalities. Among the various strategies for antimicrobial coating, electrospun fiber-based systems are particularly promising due to their intrinsic advantages, such as high porosity, high surface-area-to-volume ratio, and tunable drug-loading and release profiles. These characteristics make electrospun ultrafine fibers highly suitable for wound care applications, where they can be deposited directly onto traditional substrates (e.g., cotton gauze or commercial bandages), transforming passive dressings into active therapeutic platforms. Their ECM-like structure also promotes cell adhesion and proliferation while enabling localized delivery of antimicrobial, anti-inflammatory, or regenerative agents.

**Figure 3:**
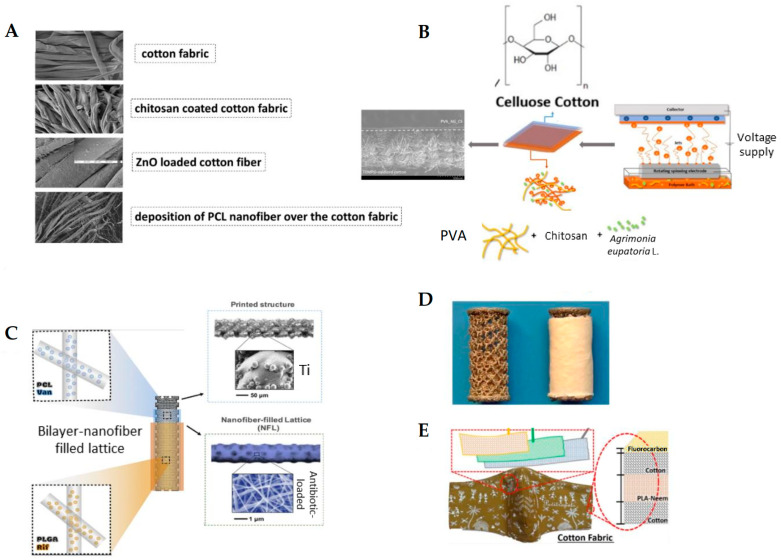
Examples of biomedical applications of antibacterial fiber coatings: (**A**) FE-SEM images of a multifunctional wound dressing fabricated by coating commercial cotton gauze with CS, ZnO nanopowder, and electrospun PCL fibers loaded with antibiotics. This composite structure enhances antibacterial activity and promotes wound healing; adapted from Ref. [162]. (**B**) Schematic of a dual-layer cotton-based wound dressing coated with PVA-CS electrospun fibers incorporating *Agrimonia eupatoria* L. extract. The bilayer configuration combines the structural support of cotton with the bioactivity of herbal-loaded electrospun fibers for advanced wound care; adapted from Ref. [168]. (**C**) Bi-layered electrospun fibers of PCL and PLGA applied to titanium implants for sustained co-delivery of rifampicin and vancomycin. This approach provides durable implant coatings that effectively prevent both early and delayed implant-associated infections; adapted from Ref. [174]. (**D**) Vancomycin-loaded collagen/hydroxyapatite (COLHA+V) fiber layers electrospun onto 3D-printed titanium implants. This coating prevented *S. epidermidis*-induced bone damage and improved osseointegration; adapted from Ref. [175]. (**E**) Schematic of a 3-layer biodegradable face mask incorporating needleless electrospun phytochemical-loaded fibers to combat viral transmission during the COVID-19 pandemic. The design includes a protective top layer, an active electrospun fiber middle layer, and a soft inner layer for comfort; adapted from Ref. [170].

A notable example is the functionalization of conventional cotton bandages with a dual-layer electrospun coating composed of CS, ZnO-NPs, and PCL ultrafine fibers loaded with diclofenac sodium (Figure 3A). In this design, CS provides broad-spectrum antimicrobial activity, ZnO-NPs offer both antibacterial effects and UV-protective properties, and the PCL fibrous mat serves as a drug reservoir for sustained anti-inflammatory action. Developed through ES, the system exhibited significant antibacterial activity against both *Staphylococcus aureus* and *Escherichia coli*, with inhibition zones exceeding 27 mm in standard disc diffusion assays. Additionally, the coating enabled regulated drug release in response to wound microenvironmental conditions, significantly reducing the risk of post-dressing infections [162,163].

In another approach, gelatin–honey fiber meshes were electrospun directly onto cotton gauze to produce bi-layered wound dressings with enhanced biocompatibility and antimicrobial potential. The incorporation of honey, up to 30% by weight, enhanced the water-repellent properties while maintaining fiber morphology and mechanical integrity. This design mimics the dual-layer architecture of human skin, combining the structural support of cotton gauze with the biological activity of the electrospun layer [164]. In a separate study, PVA ultrafine fibers incorporating methylglyoxal, an antibacterial component derived from manuka honey, were electrospun onto spun-bonded nonwoven backings. The resulting materials demonstrated strong antibacterial activity against *S. aureus* and *E. coli*, thereby validating methylglyoxal as a functional additive in bioactive wound dressings [165]. Further research was conducted by ES PVA and whey protein ultrafine fibers onto melt-blown polypropylene substrates, followed by functionalization with a citral-based Pickering emulsion stabilized by β-cyclodextrin. This bioactive coating displayed notable antibacterial activity, particularly against *E. coli*, reinforcing its potential for application in wound dressings [166]. Another study modified natural eggshell membranes with electrospun CS/PCL ultrafine fibers, forming a bi-layered scaffold that exhibited indirect antibacterial properties. The coated construct supported dermal regeneration and demonstrated robust cell integration in both in vitro and in vivo models, suggesting enhanced wound healing performance [167].

A notable strategy demonstrated by Mouro et al. [168] involved the fabrication of a dual-layer cotton-based wound dressing, in which a fibrous coating of PVA/CS loaded with *Agrimonia eupatoria* L. extract was electrospun directly onto cotton fabric (Figure 2B). Such a bilayer system effectively combined the cotton-substrate mechanical robustness with the bioactive and antimicrobial function of the herbal-infused nanofibrous layer. The PVA/CS coating facilitated the release of antibacterial agents while maintaining favorable wettability, porosity, and cytocompatibility. Microbiological assays confirmed a significant inhibition against *S. aureus* and *E. coli*, underscoring the therapeutic potential of medicinal plant-loaded ultrafine fibers in advanced wound care.

Nawalakhe et al. [169] improved fiber adhesion by plasma-treating cotton gauze prior to ES CS fibers from trifluoroacetic acid (TFA) solutions. Plasma activation introduced polar functional groups and increased surface roughness, quadrupling the peeling strength in mechanical tests. The dressing coated with the bioactive CS compound and plasma treatment showed good resistance to *E. coli* and *S. aureus* microbial agents, which emphasizes the advantage of combined wound dressings. In addition, plasma treatment with surface oxidation increased the adhesion between the substrate and CS ultrafine fibers through cross-linking. Compared to the uncoated gauze, the gauze coated with CS ultrafine fibers showed a higher percentage of wound exudate and blood absorption. A further example relies on a 3-layer biodegradable face mask incorporating needleless electrospun phytochemical-loaded fibers to combat viral transmission during the COVID-19 pandemic. The design includes a protective top layer, an active electrospun fiber middle layer, and a soft inner layer for comfort [170] (Figure 3E). These studies underscore the versatility and effectiveness of electrospun fibers as functional coatings for conventional wound dressings.

**Table 1:** Examples of applications of electrospun fiber coatings with antimicrobial activity for wound dressing.

Polymer	Solvent	Additive	Antimicrobial Properties			Fiber Properties	Application	Ref.
			Strains Tested	Methods Employed	Main Results			
PCL	Chloroform	CS, ZnO-NPs, diclofenac	*S. aureus*, *E. coli*	Disk diffusion test	Zone of bacterial inhibition: 27 mm for *S. aureus* and 32 mm for *E. coli.*	Fiber diameter = 110.08 ± 6.08 nm, drug-loaded fiber diameter = 216.36 ± 4.51 nm	Coating on commercial cotton gauze	[162,163]
Gelatin	Water	Honey	N/A	N/A	N/A	Fiber diameter = 189.2–323.7 nm	Coating on cotton gauze	[164]
PVA	Water	Methylglyoxal (MGO)	*S. aureus*, *E. coli*	Disk diffusion test	Zone of bacterial inhibition: 11.4 mm for *S. aureus* and 9.1 mm for *E. coli*, with 2.35 and 1.55 mg/cm^2^ of MGO, respectively.	Diameter of PVA fiber = 118 nm, diameter of PVA/MGO fiber = 166 nm	Coating on cotton spun-bond nonwoven	[165]
PVA, Whey Protein	Water	β-cyclodextrin (β-CD), citral	*E. coli* ATCC 25922, *S. aureus* ATCC 25923, *P. aeruginosa* ATCC 27853	Disk diffusion test	Zone of bacterial inhibition: 14 mm for *E. coli* and 12 mm for *S. aureus* with the highest β-CD/citral ratios (1:6).	Diameter = 216–330 nm	Coating on polypropylene wound dressing	[166]
CS/PCL (CP)	Formic acid/acetone	Eggshell nanofiber membrane (ESM)	*S. aureus*, *E. coli*	Disk diffusion test Plate count method	Zone of bacterial inhibition: 2.68 ± 0.21 cm^2^ against *E. coli* and 2.19 ± 0.17 cm^2^ against *S. aureus*, with complete bacterial elimination after 12 h.	Diameter = 400–700 nm, Porosity of CP-ESM after electrospinning = 85%, thickness = 15 µm	Eggshell membrane modification	[167]
PVA/CS	Water/Acetic Acid	*Agrimonia eupatoria* L. extract	*S. aureus* ATTC 6538, *P. aeruginosa* PA25	Standard Test Method for Determining the Activity of Incorporated Antimicrobial Agent(s) in Polymeric or Hydrophobic Materials (ASTM E2180-07 standard)	High inhibitory effect of PVA_AG_CS (99.17 ± 4.05% inhibition for *S. aureus* and 98.13 ± 0.88% for *P. aeruginosa*).	diameter of PVA-AG_CS fibers = 50–400 nm	Coating on cotton material	[168]
CS	TFA	Surface modified using atmospheric plasma (ICP)	*E. coli* O157 and B179, *B. cereus* B2	Plate Count Method	Reduction in bacterial count of *E. coli* from 7.47 ± 0.09 to 4.82 ± 0.06 log (CFU/mL) and *B. cereus* from 6.14 ± 0.14 to 2.80 ± 0.06 log (CFU/mL).	Not reported	Coating on cotton gauze	[169]
Keratin	Water/hydrochloric acid	Harmaline/Ginkgo Biloba	*Bacillus cereus*, *E. coli*	Bactericidal Activity Assay	Antibacterial efficacy at 94.74% against *B. cereus* and 96% against *E. coli*.	Diameter = 63–78 nm	Wound dressing/band aid	[171]
PVA/Wool Keratin	Water	Ag NP	*S. aureus*, *P. aeruginosa*	AATCC (American Association of Textile Chemists and Colorists) Test Method 147-1998 (Assessment of Textile Materials: Parallel Streak Method)	Cotton fabric coated with 0.1% Ag-NP-embedded PVA nanofibers showed very good antibacterial activity against both pathogens.	Diameter = 146.7 nm; Fiber thickness ~ 146.7 nm	The coating on cotton fabric	[172]
Hydroxypropyl-β-cyclodextrin (HP-β-CD)/Hydroxypropyl-γ-cyclodextrin (HP-γ-CD)	Water	Lawsone (2-Hydroxy-1,4-naphthoquinone)	*E. coli*, *S. aureus*	Plate Count Method	100% eradication of *E. coli* and *S. aureus* with no difference.	Diameter = 300–700 nm	Coating on cotton nonwoven	[173]

By carefully tailoring polymer compositions and incorporating diverse bioactive agents, including metallic nanoparticles, anti-inflammatory drugs, and natural antibacterial compounds, researchers have significantly improved the therapeutic efficacy of standard materials such as cotton gauze. The integration of electrospun fibers and other functional coatings into commercial wound dressings marks a transformative step forward in modern wound care, offering comprehensive solutions for the management of infected wounds, chronic ulcers, and complex skin injuries. These advancements facilitate the development of multifunctional wound dressings that are not only cost-effective and scalable but also capable of delivering targeted, sustained, and stimuli-responsive treatment in clinical settings.

### 5.2. Fibrous-Based Coating for Implants

Implanted medical devices, including orthopedic implants, cardiovascular stents, dental implants, and many others, are susceptible to device-associated infections. These infections often result in implant failure, posing serious health risks and significant economic burdens for healthcare systems. Figure 3C,D also shows applicative examples of medicated electrospun polymer coatings on bone implants. Bi-layered electrospun coatings were applied to titanium implants and demonstrated sustained co-delivery of antibiotics [174] (Figure 3C), whereas vancomycin-loaded collagen/hydroxyapatite fibrous layers were electrospun onto 3D-printed titanium implants to combat infection and improve osseointegration [175] (Figure 3D). In fact, despite improvements in clinical protocols, effective biocidal treatment for revision surgeries remains a challenge [51,176,177,178]. The standard approach involves surgical removal and replacement of the infected implant, which itself carries a risk of further complications. The primary cause of implant-associated infections is biofilm formation, which hinders antibiotic penetration and facilitates colonization by antibiotic-resistant microorganisms [179]. To address this issue, antibacterial surface coatings have emerged as a strategy to prevent biofilm formation and bacterial adhesion. Antibacterial coatings are generally classified based on their mode of action. Bacteria-repellent coatings, also known as anti-adhesive coatings, function by preventing the initial adhesion of bacteria to the implant surface. However, a significant limitation of these coatings is that they may also inhibit the adhesion of host cells, potentially impairing tissue integration and osseointegration [180]. On the other hand, bactericidal coatings are designed to actively kill bacteria, typically through the release of antibiotics or antimicrobial ions. While effective in reducing bacterial load, these coatings may induce toxicity due to a rapid “burst” release of the active agents and often lack a sustained and controlled release profile, which limits their long-term efficacy [181,182].

An optimal implant surface must balance biocompatibility (to promote osseointegration and host tissue integration) and antibacterial activity (to prevent colonization) [183,184]. This is a challenging goal since mechanisms facilitating host-cell adhesion may also support bacterial attachment [184]. Recent research has aimed to combine repellent and bactericidal functionalities into hybrid coatings [176]. Yet, bacterial adhesion via protein adsorption remains a problem, and coatings with high drug-loading capacity, tunable release profiles, and long-lasting antimicrobial action are still needed [170]. Electrospun fiber coatings have garnered significant interest for use in antimicrobial implants due to their large surface area, tunable porosity, and capacity to incorporate a wide range of therapeutic agents [185,186]. These fiber-based coatings offer several advantages. They enable controlled and sustained release of drugs and nanoparticles, enhance tissue integration and overall implant biocompatibility, and can be customized through various polymeric blends and environment-friendly (green) solvents [51,175,187,188]. Various electrospun polymer-based coatings have been developed for orthopedic and dental implants, as summarized in Table 2. Commonly used polymers include PCL, PLGA, CS, and their blends. The choice of solvent plays a critical role in polymer dissolution and fiber formation, influencing coating morphology and therefore drug release kinetics. While solvents such as HFIP and trifluoroethanol (TFE) are widely effective for ES, their use raises significant toxicity concerns [189,190]. To provide antimicrobial functionality, these coatings often incorporate antibacterial agents such as Ag-NPs, which provide broad-spectrum activity but carry potential risks of cytotoxicity, bioaccumulation, and oxidative stress [189,190]. Natural antimicrobial agents like plant extracts, including thyme and henna, are also used for their biocompatibility and low resistance potential. Understanding biodistribution, long-term effects, and mechanisms of action is crucial for ensuring safe biomedical applications of agents due to the association of chronic exposure to organ damage and altered metabolic pathways [191,192]. Several studies have demonstrated the effectiveness of CS/PEO blends dissolved in acetic acid, a biobased and environmentally friendly solvent. These have been combined with antimicrobial agents like cefepime [193], which showed sustained drug release for up to 16 days with minimal initial burst release, and vancomycin [194], which achieved both burst and sustained release profiles when a PLGA coating was applied. Similarly, coatings incorporating henna or thyme extracts exhibited higher antibacterial activity against Gram-positive bacteria [195]. Additional formulations include CS/PEO combined with bioactive glass particles to promote osteoconductivity and bacteriostatic effects [196], as well as blends of PEO with carboxymethylcellulose (CMC) loaded with clindamycin, in which cross-linking steps enhanced long-term stability for potential implant coating applications [197].

Alternative fiber-based approaches have also been explored to enhance antimicrobial efficacy. TiO_2_ ultrafine fibers synthesized using a combination of sol–gel processing and ES demonstrated dose-dependent antibiofilm activity, with particularly strong effects against *P. aeruginosa* [198]. Similarly, Ag-NPs-loaded ultrafine fibers based on 2-hydroxypropyl-β-cyclodextrin (HP-β-CD), produced using either dimethylformamide (DMF) or water as solvents, showed significant inhibition of *E. coli* and *S. aureus*. The fiber diameter and the choice of solvent were found to influence the antibacterial performance, with DMF-based fibers generally exhibiting smaller diameters and stronger antimicrobial effects [199]. As anticipated earlier, Jahanmard et al. [174] developed a bilayer fiber coating system for orthopedic implants using electrospun PCL and PLGA layers loaded with vancomycin and rifampicin (Figure 3C). This dual-drug configuration achieved a synergistic antibacterial effect against *S. aureus* and *S. epidermidis*, effectively preventing both early and delayed post-surgical infections. The electrospun fiber-filled lattice coating adhered firmly to titanium implants and exhibited a sustained release profile, minimizing burst release and maximizing localized therapeutic efficacy. This approach highlights the potential of multifunctional electrospun coatings for infection-resistant implantable devices in complex wound environments.

Suchý et al. [175] designed an advanced antibacterial implant coating by ES vancomycin-loaded collagen/hydroxyapatite (COLHA+V) fibers onto 3D-printed titanium implants (Figure 3D). This multilayered system combined the bioactivity of collagen and osteoconductivity of hydroxyapatite with the potent antimicrobial properties of vancomycin. The coating significantly inhibited *S. epidermidis* biofilm formation and preserved bone structure in infected murine femoral defects. Moreover, the COLHA+V layer promoted osseointegration, indicating its dual function in both infection prevention and bone tissue regeneration. This approach highlights the clinical potential of electrospun bioactive fiber coatings for orthopedic and dental implants. Despite significant progress, several challenges remain for the future development of antimicrobial implant coatings. Most current research has focused primarily on orthopedic and vascular implants, leaving other implant types relatively underexplored. Additionally, the lack of standardized testing protocols for evaluating antimicrobial efficacy hinders meaningful comparison between studies. To advance the field, future efforts must prioritize the development of sustainable coatings using green solvents and biodegradable polymers. Comparative studies conducted under unified, standardized testing conditions are essential to assess and validate coating performance. Furthermore, addressing the growing issue of antibiotic resistance and minimizing environmental contamination from drug leaching must be central considerations in the design of next-generation antimicrobial coatings.

### 5.3. Fbrous Coatings for Surgical Drapes, Gowns, and Facemasks

Surgical drapes and gowns are critical components of infection control in clinical settings, serving to maintain a sterile environment and prevent the transmission of pathogens during surgical procedures. Recent reviews emphasize the importance of incorporating antimicrobial properties directly onto these garments, both reusable and disposable, to extend their lifespan, reduce environmental impact, and address real-world challenges posed by hospital-acquired infections [209]. Among the various approaches explored, ES has emerged as a highly effective technique for fabricating polymeric nano/ultrafine fiber coatings directly onto textile surfaces (Table 3). These electrospun fiber coatings offer significant advantages over conventional chemical treatments, including enhanced durability, tunable porosity, high surface area, and the ability to incorporate antimicrobial agents such as metal nanoparticles or biocidal polymers. The resulting nanofibrous layers serve as multifunctional barriers that resist microbial adhesion, inhibit bacterial growth, and maintain essential properties such as breathability, mechanical integrity, and wearer comfort [210]. Several recent studies have successfully demonstrated the application of electrospun coatings on surgical drapes and gowns to enhance their antimicrobial performance. In one example, ZnO-loaded PVA fibers were electrospun onto gown fabrics, producing bead-free fiber mats that exhibited clear zones of inhibition against both Gram-positive and Gram-negative bacteria. Importantly, these antimicrobial coatings maintained the tensile strength and hydrophilicity of the original fabric, ensuring wearer comfort and durability [211]. In response to the heightened need for protective measures following the COVID-19 pandemic, another study employed polyacrylonitrile (PAN)/ZnO fibers containing 5 w% of the antiviral Viroblock formulation. These electrospun fibers were applied directly onto textile substrates, resulting in hybrid mats capable of achieving over 92% bacterial inactivation against *S. aureus* and *P. aeruginosa*, along with a 37% reduction in viral titer for enveloped viruses. These results highlight their dual protective functionality against bacterial and viral threats [212].

In a further advancement, electrospun fibers made of PVA, which is potentially biodegradable in soil environments under the right microbial and environmental conditions, embedded with ZnO-NPs or copper oxide (CuO)-NPs were developed as antimicrobial coatings for gown materials. Both dynamic and static contact assays confirmed the effectiveness of ZnO-enriched membranes in inactivating SARS-CoV-2 surrogate and eliminating multidrug-resistant bacterial strains such as methicillin-resistant *S. aureus* (MRSA) and methicillin-resistant *S. epidermidis* (MRSE). These coatings also preserved essential properties such as breathability and mechanical integrity, making them suitable for real-world clinical applications [213]. Electrospun fiber technologies have also been explored for antimicrobial facemasks. A notable example by Patil et al. involved the use of needleless ES to fabricate a 3-layer biodegradable facemask composed of a cotton/PLA/cotton structure (Figure 3E). The PLA nanofibrous layer, electrospun from dichloromethane (DCM) solution, was embedded with phytochemicals extracted from *Azadirachta Indica* and *Eucalyptus Citriodora*. This configuration enhanced bacterial filtration efficiency by increasing surface area and forming a dense, interconnected network capable of capturing particulate matter, aerosols, and bacteria deep within the filtration matrix [170].

These studies collectively demonstrate that electrospun antimicrobial coatings can be effectively tailored to integrate with existing medical textiles, significantly enhancing infection control, UV protection, and self-sterilizing capabilities. Importantly, these enhancements are achieved without compromising critical performance attributes, including breathability, tensile strength, and wearer comfort, qualities essential for surgical drapes and gowns.

The application of electrospun fiber coatings represents a novel and promising strategy for reinforcing antimicrobial barriers in healthcare settings. By enabling the direct incorporation of functional agents at the surface–pathogen interface, these coatings maintain the structural and functional integrity of the base fabric while offering high-performance, sustainable, and safe protective solutions.

### 5.4. Fibrous Coating for Catheters and Ureteral Stents

Catheters and ureteral stents are critical medical devices widely used in clinical settings for vascular access, enteral nutrition, and urinary tract management. Catheters, defined as hollow tubes, are typically categorized into vascular (central or peripheral) and enteral types based on their application [214]. Ureteral stents, on the other hand, are used to manage a variety of urinary tract conditions such as obstructions, strictures, nephrolithotomy, and tumors by facilitating urine flow [215]. Despite their utility, prolonged use of these devices often leads to catheter-associated urinary tract infections (CAUTIs), among the most common and difficult nosocomial infections in healthcare. These infections not only compromise patients’ comfort but also contribute to increased morbidity, mortality, and healthcare costs [216].

These infections arise from microbial adhesion, colonization, and subsequent biofilm formation on the device surface. The ideal stent must therefore exhibit properties such as biocompatibility, mechanical stability, biodegradability, resistance to irritation, encrustation, migration, and biofilm formation while maintaining urine flow and the patient’s comfort. Additionally, features like radiopacity, elasticity, and cost-effectiveness are essential for optimal clinical performance [217]. To address these challenges, ES has emerged as a promising technique for fabricating or coating urinary stents. ES enables the production of porous and tubular structures that mimic the extracellular matrix, improve tissue integration, and enable localized drug delivery. By utilizing biopolymers that possess suitable viscosity and spinnability, it is possible to further enhance ES potential for device modification [218]. However, studies on the use of electrospun coatings for ureteral stents remain limited. Key factors in the coating process rely on the adhesion between the electrospun polymer fibers and the substrates and on the varying shapes of the device to be coated, which must be assessed. In fact, diverse outcomes can be experienced by ES on the same polymer surface, as well as on different polymer surfaces, due to the electric conductivity, chemical compatibility, and interaction with the electric field generated by the ES process.

Table 4 offers a synopsis of extant research in this domain. Notable research in this area includes the work by Korniienko et al., who electrospun CS fibers using a trifluoroacetic acid (TFA)/DCM solvent blend. The resulting meshes demonstrated bacteriostatic effects against planktonic bacteria and biofilms, indicating potential for antimicrobial applications [219]. Another study by Salih et al. developed a PLGA/CS fiber blend embedded with Zn/Al-layered double hydroxide (LDH) NPs using HFIP as a solvent. Zn^2+^ ions provided antibacterial functionality, while 0.5 w% CS further enhanced bioactivity. The electrospun composite meshes showed effective antibacterial activity against *S. aureus* and *E. coli*, along with adequate mechanical strength and good biocompatibility [220].

Another advancement involved the fabrication of fully biodegradable electrospun stents using PLGA integrated with a silver@graphdiyne (Ag@GDY) nanocomposite. These constructs, created using an ES-based method, replicated the porous architecture of natural tissues. The incorporation of Ag@GDY endowed the stents with antimicrobial properties, reducing both bacterial adhesion and biofilm formation. The electrospun structure supported gradual degradation, showing a decrease in toughness as well as an increase in strength over time. In vivo and cellular tests confirmed moderate biocompatibility and demonstrated the stent’s potential as an alternative to traditional PU devices for urinary applications [221].

While electrospun coatings for ureteral stents have shown considerable promise in enhancing antimicrobial performance, tissue compatibility, and mechanical integrity, further research is necessary. Expanding studies in this field could lead to next-generation biodegradable and infection-resistant urinary stents that effectively address the persistent clinical challenges associated with CAUTIs and stent-related complications.

## 6. Innovations in Green ES for Antibacterial Applications

**Figure 4:**
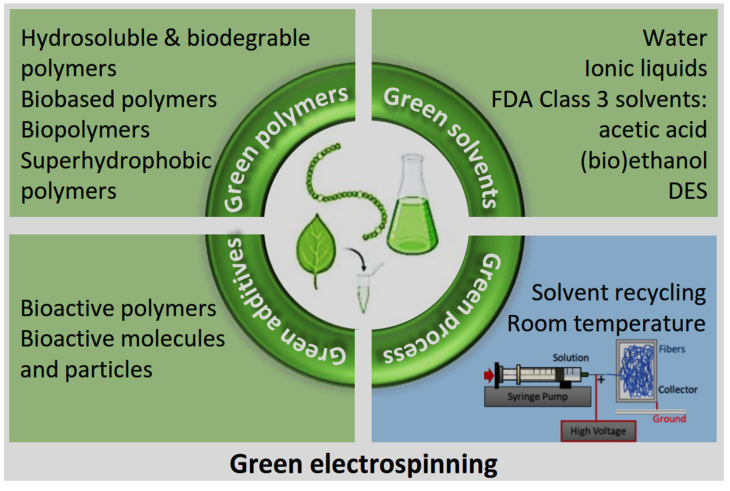
Sustainable approaches in green ES: polymers, solvents, additives, and energy reduction strategies (created by the authors).

Recent advancements in green ES have significantly transformed the development of antibacterial nanofibrous materials by emphasizing the use of eco-friendly solvents, renewable biopolymers, and naturally derived antimicrobial agents. These innovations address environmental concerns, enhance biocompatibility, and reduce the overall carbon footprint of biomedical materials (Figure 4). A diverse range of renewable biopolymers, including chitin, cellulose, gellan and Arabic gums, collagen, gelatin, alginate, lignin, and silk, have been explored for their intrinsic biodegradability and sustainability [221]. These materials are typically classified based on their chemical composition into protein-based (e.g., silk, collagen) and polysaccharide-based (e.g., cellulose, CS, dextran) [222,223].

CS, derived from chitin, the second most abundant biopolymer after cellulose, is particularly notable for its biodegradability, biocompatibility, antimicrobial activity, and wound-healing properties [224]. Despite their desirable biological properties, natural polymers like CS and SF face challenges in ES due to their poor solubility and limited spinnability in aqueous solutions. To address this, they are often blended with synthetic polymers such as PVA or PEO to facilitate fiber formation while retaining functional characteristics [224]. The use of green solvents, such as acetic acid and formic acid, may enable the dissolution and processing of these biopolymers. Moreover, additives like surfactants, salts, and co-solvents have been employed to reduce surface tension and improve the spinnability of ES solutions [225]. A novel development in this area is the use of D-limonene, a citrus-derived monoterpene, as a green solvent alternative for ES [226]. These eco-friendly solvents not only reduce environmental toxicity but also enable the fabrication of ultrafine fibers suitable for biomedical use. Avossa et al. provided a comprehensive review of green ES, highlighting methods for producing polymeric fibers using solution, emulsion, suspension, and in situ cross-linking approaches. The study also classified solvents based on their environmental impact and outlined sustainable strategies for integrating natural polymers with benign solvents, thereby improving both the ecological and economic performance of ES processes [92].

The functionalization of electrospun fibers with antibacterial agents remains a critical aspect of green ES. However, traditional additives such as antibiotics and metallic nanoparticles pose environmental and health risks. In contrast, phytochemicals, namely, bioactive compounds derived from plants, offer sustainable, low-toxicity alternatives. These natural compounds possess inherent antimicrobial and medicinal properties with minimal side effects [227,228]. Adamu et al. emphasized the use of plant-based extracts in wound healing applications, displaying their promise as green antibacterial agents. Despite these advantages, research on electrospun fibers incorporating plant extracts remains limited. The earliest example is the incorporation of *Curcuma longa* (turmeric) extract into cellulose acetate fibers via ES, which demonstrated antibacterial potential [229].

Another emerging area is the development of stimuli-responsive smart electrospun fibers, which exhibit antimicrobial activity triggered by external stimuli such as pH, temperature, or light. Mercante et al. reviewed the progress in this area, underscoring its potential for advanced antibacterial applications [111]. Similarly, Chan et al. explored the biomedical potential of smart electrospun fibers in wound healing, cancer therapy, and tissue engineering, although challenges persist in translating these materials into clinical use [230].

Finally, superhydrophobic surface behavior, as obtained via superimposing electrospun fiber layers made of hydrophobic polymers, has been reported as a successful strategy to limit bacterial adhesion and therefore their survival [22]. Indeed, by controlling the fiber orientation and the generated pore size during ES deposition, fibrous poly(vinylidene fluoride-co-trifluoroethylene) (PVDF-TrFE) multilayers (8–10 layers) were obtained, acting under the Cassie–Baxter regimen, thus behaving as a superhydrophobic surface, which in turn discouraged adhesion, infiltration, and biofilm formation of *S. epidermidis*, *E. coli,* and *P. aeruginosa* [207].

## 7. Challenges and Future Perspectives

Microbial contamination remains a major concern in healthcare settings, threatening patient safety and complicating infection control. Antimicrobial resistance and hospital-acquired infections continue to drive the demand for innovative, antibiotic-free antibacterial strategies. Advances in biomaterials and nanotechnology, particularly the integration of bio-nanotechnology with natural and synthetic polymers, offer promising solutions to reduce these risks [231,232]. Biopolymer-based fibers with inherent antimicrobial activity are increasingly being explored for diverse biomedical applications, including wound dressings, implant coatings, surgical drapes, and protective textiles. These fibers present an environmentally responsible and potentially more sustainable alternative to conventional antibacterial agents. However, despite significant laboratory successes, several critical challenges hinder their broad clinical and commercial implementation [233].

### 7.1. Scalability and Commercial Translation

One of the major challenges is the scalability and commercial viability of electrospun nanofibrous materials. While laboratory-scale techniques, such as ES, provide precise control over fiber morphology and composition, the transition from lab- to industrial-scale production remains technically and economically demanding. The production processes are often limited by low throughput, high energy consumption, and the need for precise environmental control [234]. Additionally, cost-effectiveness continues to pose a challenge when evaluating advanced nanofibrous coatings in comparison to traditional antibacterial agents. Without affordable large-scale manufacturing, the adoption of these technologies in mainstream medical products may be restricted [235]. To bridge this gap, research must focus on optimizing scalable fabrication methods, such as needleless ES or centrifugal spinning, as well as exploring automation and continuous production systems.

### 7.2. Emerging Trends and Strategic Opportunities

Looking ahead, several emerging trends are likely to significantly influence the development of next-generation antibacterial nano/ultrafine fiber technologies. Key directions move towards the development of smart antibacterial coatings, which integrate stimuli-responsive functionalities, such as pH, temperature, or light-triggered antimicrobial release, to enhance targeted efficacy and minimize side effects [28], and towards superhydrophobic fiber coatings [207], made of biobased hydrophobic polymers. In parallel, the rise of personalized medicine and point-of-care diagnostics is creating opportunities to tailor antibacterial coatings to individual patient needs and medical conditions, thereby improving treatment specificity and therapeutic outcomes [236]. Simultaneously, the exploration of advanced biomaterials and composite systems, including novel polymers, bioactive compounds, and hybrid nanostructures, is enabling the design of nano/ultrafine fibers with enhanced multifunctionality, mechanical stability, and biocompatibility [237]. To ensure the successful translation of these innovations into clinical practice, interdisciplinary collaboration among materials scientists, biomedical engineers, microbiologists, clinicians, and industry stakeholders is critical. Such collaborations can facilitate the resolution of key challenges related to regulatory approval, safety, and performance requirements [237]. By overcoming scalability limitations and integrating these emerging technologies, researchers can accelerate the transition of antibacterial ultrafine fiber coatings from laboratory development to practical, real-world healthcare applications, ultimately offering safer, more effective, and environmentally sustainable solutions for infection prevention [238].

## 8. Conclusions

Polymer-based fiber coatings represent a promising strategy for antibacterial applications in healthcare, offering key advantages such as high surface area, tunable porosity, and controlled release capabilities. However, conventional approaches that rely on petrochemical-derived polymers and synthetic antibacterial agents are facing growing concerns, including microbial resistance, cytotoxicity, and environmental burden. These challenges have catalyzed a transition towards more sustainable alternatives, particularly biopolymer-based functionalized fibers and natural antimicrobial agents, which can provide effective antimicrobial action while minimizing adverse effects on human health and the environment. This review has outlined the fundamental principles of fiber formation, fabrication methods, and sustainable strategies for green polymer-based fiber synthesis, with a focus on eco-friendly materials, green solvents, and biodegradability. The mechanisms underlying antibacterial activity, along with factors influencing fiber performance, were explored in detail. In addition, key biomedical applications, particularly as coatings for wound dressings, bone implants, surgical textiles, and medical devices, highlight the versatile role of these materials in enhancing infection control, promoting tissue healing, and contributing to more sustainable clinical practice. Despite notable advancements, several challenges remain. Future research should prioritize optimizing surface functionalization methods, improving long-term antibacterial efficacy, and overcoming barriers related to scalability and cost-efficiency for clinical translation. Additionally, the development of smart coatings with tunable, time-dependent release profiles, or acting by physical and electrostatic methods, combined with robust interdisciplinary collaboration, will be vital for bridging the gap between laboratory innovation and real-world healthcare solutions. These efforts will ultimately support the development of next-generation antibacterial fiber coatings that are safe, effective, and environmentally sustainable.

## Figures and Tables

**Table 2:** Examples of applications of electrospun fiber coatings with antimicrobial activity for biomedical implants.

Polymers	Solvent	Additives	Antimicrobial Properties			Application	Fiber Properties	Ref.
			Strains Tested	Methods Employed	Main Results			
PLGA/PCL	TFE	Rifampicin/vancomycin hydrochloride	*S. aureus* ATCC 49230 (originally isolated from an osteomyelitis patient)	Disk diffusion test at 1, 14, 28, and 42 days	Rif-loaded bilayer showed strongest and sustained antibacterial activity up to 42 days. Van-loaded active at day 1 only.	Metallic implantable devices	Not reported	[174]
PLGA/PCL	HFIP/ DCM/2-propanol	Linezolid, Vancomycin, daptomycin	*S. aureus* Xen36 (clinical bacteremia isolate ATCC 49525)	Disk diffusion test CFU standard curve	The greatest zone of inhibition (ZOI) was observed with the composite coating containing different antibiotics, whereas the single-antibiotic coatings showed the smallest ZOIs.	Orthopedic implants	Thickness ∼ 10–100 μm	[177]
PVA-co-PE	Isopropanol/water	Photoactive agent (BPTCD), zwitterionic monomer (SBMA)	*E. coli* O157: H7	Plate Count Method Morphological assay ROS producing assay	Reduction of 6 log CFU in 1 h under UVA producing ^·^OH. The materials maintain biocidal activity for 5 cycles and cause obvious structural deformations in bacterial cells.	Medical applications	Diameter = 522 nm	[185]
PLGA/PEO	TFE	Gentamicin sulfate	*S. aureus* ATCC 29213	Agar Diffusion Test Bacterial Adhesion Test	The gentamicin-coated titanium implants exhibited a persistent antibacterial efficacy for 1 week and significantly reduced the adhesion of the *S. aureus* compared with bare titanium implants in vitro	Orthopedic titanium implants	Diameter = 705 ± 122 nm	[187]
Collagen	PBS/ethanol	hydroxyapatite	clinical isolate of *Staphylococcus epidermidis*	MIC (EUCAST guidelines) Plate Count Method Biofilm Formation (Christensen method)	Initial bacterial load (10^4^–10^6^ CFU/mL) reduced to 10^3^ CFU/mL in 3/16 implants after 7 days in vivo.	Orthopedic implants	N/A	[188]
PLLA	DCM	Akermanite (AKT); doxycycline (DOXY)	*S. aureus* ATCC 12600, *E. coli* ATCC 9637	Disk diffusion test sensitivity and liquid medium micro dilution experiments	Inhibition zones up to 4.08 ± 0.30 mm against *S. aureus* and >98% bacterial inhibition in liquid at 10 wt% DOXY. Dose-dependent effect on *E. coli.*	Orthopedic applications	Diameter = (300–350 nm)	[189]
PCL	Chloroform/methanol	Hydroxyapatite	*S. aureus* NCIM: 5021	Optical Density (OD) Measurement at 600 nm	High reduction in adhesion (high with PCL, low with HA).	Orthopedic implants	Diameter ∼ 100 nm	[190]
CE	DMF/acetone	Ag-NPs	*E. coli* NBRC 3301, *S. aureus* NBRC 12732	Disc diffusion assay (Kirby Bauer method) by area of inhibition zone;	Exhibition of high activity against *S. aureus* and *E. coli,* respectively, with 347.13 mm^2^ and 269.96 mm^2^ area of inhibition zone.	Antibacterial applications	N/A	[191]
PCL/CS	Acetic/formic acid	Nano-CaTiO_3_ (CA1), Nano-BaTiO_3_ (BA1)	*S. aureus*, *Streptococcus mutans*	Antibacterial inhibition zone test (spread plate technique)	Significant inhibitory effects against *S. aureus* and *S*. *mutans*, with inhibition zones of 17 mm and 20 mm for CA1, and 18 mm and 17 mm for BA1, respectively.	Orthodontic and orthopedic applications	Fiber diameter for (CA1) = 162 ± 62 nm and an average size porosity of 1360 ± 5242 nm	[192]
CS/PEO	Acetic acid	Cefepime SiO_2_	*S. aureus* ATCC 6538, *S. epidermidis*, *E. coli* ETEC ATCC 35401	Disk diffusion test	Inhibitory effect with 25 and 40 mm against *S. aureus* and *E. coli*, respectively.	Orthopedic implants	diameter = 171.745 nm	[193]
CS/PVA CS/PEO PLGA	Acetic acid	Vancomycin	Sustained antibiotic release, biofilm prevention	Kinetics of the release	N/A	Vascular grafts	Diameter of CS/PVA = 164.1 ± 65.34 nm; diameter of CS/PEO = 240 ± 50.81 nm	[194]
CS/PEO	Acetic acid	Henna and Thyme Leaves Extract	*S. aureus*, *E. coli*.	Disk diffusion test	Inhibition zones reaching up to 5.3 ± 0.2 cm for *E. coli* and 5.0 ± 0.1 cm for *S. aureus*.	Orthopedic implants	Not reported	[195]
CS/PEO	Acetic acid and water	Bioactive Glass (BG)	*S. epidermidis* ATCCTM 14990	Microbial Viability Assay Plate Count Method Fluorescence Microscope	Significantly reducing the growth (effective at 48 h). Bacterial lysis and low proliferation.	dental and orthopedic applications	Diameter = 493.21 ± 77.11 nm)	[196]
PEO/CMC	Ethanol	Clindamycin hydrochloride monohydrate	*Streptococcus* sp., *Staphylococcus* sp., *Pneumococcus* sp.	In Vitro Release Test	N/A	Orthopedic implants	Diameter = 521 ± 115 nm	[197]
polyvinylpyrrolidone (PVP)	Acetic acid/ethanol	TiO_2_	*P. aeruginosa* PAO1, methicillin resistant *S. aureus* (MRSA) ATCC 33591	Minimum Inhibitory and Minimum Bactericidal Concentration (MIC/MBC)	Significant antibacterial properties (MIC/MBC: 6/12 mg/mL for MRSA, 3/6 mg/mL for *P. aeruginosa*).	Various biomedical applications	Diameter = 80–600 nm; thickness ~ 400 nm	[198]
HP-β-CD	DMF, H_2_O	Ag-NPs	*E. coli* RSHM 888, *S. aureus* RSHM 96090/07035 (ATCC 25923)	Disk diffusion test	Diameters of inhibition zones: 1.07±0.038 and 1.16 cm for *E. coli* and *S. aureus*, respectively.	Antibacterial application	diameter ~ 207–440 nm	[199]
PCL	HFIP	Au-NPs Norfloxacin, Nystatin, Gentamicin	*S. aureus* ATCC 29213, *E. coli* ATCC 10536, methicillin-susceptible *S. aureus* (MSSA MFBF 10663), methicillin-resistant *S. aureus* (MRSA MFBF 10679), clinical isolates (Collection of Univ. of Zagreb, KR)	Disk diffusion test Serial Microdilution Broth Assay Minimum Inhibitory Concentration (MIC) Assay	No antimicrobial activity was observed for Au nanoparticles (10 nm, 20 ppm), while standard antibiotics (Norfloxacin, Nystatin, Gentamicin) produced inhibition zones ranging from 17 to 23 mm against *S. aureus*, *E. coli*, and *C. albicans.*	Musculoskeletal medical implants	N/A	[200]
PCL/CS/PEO	DCM, DMF, acetic acid	Ag-NPs	*E. coli*-ATCC 25922, *S. aureus*-ATCC 25923	Bacterial viability through plate count (CFU/mL) and OD_600_ measurement Cytotoxicity test	Reduction in bacterial viability depending on concentration of AgNPs (more in *E. coli*), with maximum effect with exposed electromagnetic waves. Increase in membrane damage and cell lysis (release of LDH, proteins, and nucleic acids).	Vascular stents	Diameter = 100 nm	[201]
Gelatin	TFE	Ag-NPs	*S. aureus* ATCC-6538, *E. coli* ATCC-8739.	Minimum inhibitory concentrations (MICs) Bacterial colony counts	Significant antimicrobial activity at 48 h, showing complete (99.99%) inhibition of bacterial colony counts at 24 and 48 h.	Dental implants	Diameters of individual fibers = 300 to 400 nm	[202]
PLLA/gelatin	TFE	Hydroxyapatite nanowire Ag-NPs	*E. coli*, *S. aureus*	Disk diffusion test Plate Count Method	Elimination of *E. coli* by 100% and reduction *S. aureus* by 85–96%. The formulation with Ag and HA shows 100% antibacterial activity on both strains.	Orthopedic implants	Diameter = 221–262 nm	[203]
PLA/PCL/Gelatin	HFP	Tetracycline hydrochloride	Gram-negative anaerobic bacteria: *Porphyromonas gingivalis (Pg*, ATCC 33277), *Fusobacterium nucleatum* (*Fn*, ATCC 10953), *Prevotella intermedia (Pi*, ATCC 25611), *Aggregatibacter actinomycetemcomitans* (*Aa*, ATCC 33384)	Biofilm inhibition assay	Reduction in biofilm formation of all strains with a dose-dependent effect (max. at 25%), with *Pi* the most resistance but still sensitive.	Dental implant	Diameter = 172–393 nm	[204]
Cellulose acetate	Acetone	Polydiallyldimethylammonium chloride (pDADMAC)	*E. coli* K12	OD reading at 600 nm	Inactivation of *E. coli* up to 97.2% ± 4% by pDADMAC-functionalized nanofiber	Antimicrobial applications	Diameter = 0.85 ± 0.22 µm Bulk thickness = 42.4 ± 12 μm	[205]
PLGA	Trifluoroethanol	Vancomycin	*S. aureus* ATCC 29213	Disk diffusion test; In Vivo Test	Strong in vitro antimicrobial activity, with a peak inhibition zone of 12.7 ± 0.4 mm on day 1 and sustained antibacterial efficacy via biphasic release over 28 days. No deaths in the vancomycin group	Orthopedic applications	Diameter = 728–983 nm	[206]
PVDF-TrFE	MEK	-	*E. coli* 25922, *P. aeruginosa* PAO1, *S. epidermidis* 35984	Bacterial adhesion assay	Reduction in bacterial adhesion by ~69% compared to smooth films, against both *E. coli* and *P. aeruginosa*. Reduction against *S. epidermidis* adhesion without statistical significance.	Biomedical surface coatings	Diameter = 0.97 ± 0.34 μm; mean pore size = 11.6 μm surface porosity = 58% size range = 0.32–1.65 μm	[207]
PLA	chloroform/DMF	Curcumin, 45S5 BG, mesoporous silica nanoparticles (MSNs)	*E. coli*, *S. aureus*	Plate Count Method Disk diffusion test	For *S. aureus*: Nanofiber treatment reduced CFU from 0.14 × 10^15^ (control) to 0.45 × 10^11^ at the highest concentration (S3). For *E. coli*: CFU decreased from 0.4 × 10^14^ (control) to 0.3 × 10^0^ with S3 nanofiber treatment.	Orthopedics and dentistry implants	Thickness = 10 to 15 μm; pore diameters = 0.5 to 50 μm that plain diameter of plain PLA fiber = 461 nm, diameter of curcumin-loaded fiber = 350 nm	[208]

**Table 3:** Examples of applications of electrospun fiber coatings with antimicrobial activity for surgical drapes and gowns.

Polymer	Solvent	Additives	Antimicrobial Properties			Fiber Properties	Application	Ref.
			Strains Tested	Methods Employed	Main Results			
PLA	DCM	Phytochemical herbal extracts	*S. aureus* ATCC 6538	Bacterial Filtration Efficiency (BFE) test by the IS 16288:2014 standard	Bacterial filtration efficiency of 97.9%.	Diameter = 8 ± 0.2 µm, mean pore size of 20.1429 µm	Biodegradable face mask	[170]
PVA	H_2_O	ZnO-NPs	*S. aureus*, *E. coli*	Disk diffusion test	Zone of inhibition increases with concentrations of ZnO-NPs (9% the highest effect) against *E. coli* and *S. aureus*.	Diameter = 393 ± 57	Coating for surgical gowns	[211]
PAN	DMF (or DMAc)	ZnO-NPs + Viroblock	*S. aureus*, *P. aeruginosa*	Quantitative antibacterial analysis according to AATCC-100 standard	92.59% reduction in *S. aureus* and 88.64% of *P. aeruginosa.*	Diameter = 127 ± 24.8 nm for pristine PAN, 171 ± 29.88 for 5% loaded ZnO/PAN	Nanocomposite for PPE masks and gowns	[212]
PVA	H_2_O	ZnO-NPs, CuO-NPs	*S. aureus* ATCC 6538, *Klebsiella pneumoniae* ATCC 4352, Methicillin-resistant *S. aureus* (MRSA), Methicillin-resistant *S. epidermidis* (MRSE), *P. aeruginosa*, *K. pneumoniae* (from Alexandria University hospital, Egypt)	Disk diffusion test ASTM E 2149-01 standard test	Superiority of the ZnO-loaded nanofibers over the CuO-loaded fibers. Different degrees of growth inhibition depending on the bacterial species and ZnO concentration.	Diameter = 200–250 nm	Biodegradable membranes for PPE and gowns	[213]

**Table 4:** Examples of applications of electrospun fiber coatings with antimicrobial activity for catheters and urinary stents.

Polymer	Solvent	Additives	Antimicrobial Properties			Fiber Properties	Application	Ref.
			Strains Tested	Methods Employed	Main Results			
CS	TFA/DCM	-	*S. aureus* B 918, *E. coli* B 926	Dynamic Contact Antibacterial Assay (Spread Plate CFU Count Method); Resazurin (AlamarBlue) Assay for Biofilm Viability	Inhibition of bacterial growth until 6 h (higher activity against *E. coli*), no more activity after 8 h. Ch-FA/DCM 9:1 shows better biofilm eradication; *S. aureus* is less sensitive to treatment with Ch-TFA/DCM 7:3 + NaOH (*p* < 0.001), *E. coli* shows a similar trend (*p* < 0.01).	Diameter = 0.18 ± 0.009 µm; Porosity = 9.48%	Antiadhesive capability	[219]
PLGA/CS	HFIP	LDH-NPs + Zn/Al cation	*E. coli*, *S. aureus*	Inhibition zone measurement	The addition of LDH and chitosan in PLGA increases antibacterial efficacy, with inhibition zones up to 14.1 ± 1.2 mm	Diameter = 300–600 nm	Ureteral Stent	[220]

## Data Availability

No new data were created or analyzed in this study. Data sharing is not applicable to this article.

## Abbreviations

The following abbreviations are used in this manuscript:

Ag@GDY	silver@graphdiyne
BC	Bacterial cellulose
BmimAc	1-butyl-3-methylimidazolium acetate
CAUTI	Catheter-Associated Urinary Tract Infection
CMC	Carboxymethyl Cellulose
COLHA+V	vancomycin-loade collagen/hydroxyapatite
COVID-19	Coronavirus Disease 2019
CS	Chitosan
DCM	Dichloromethane
DES	Deep eutectic solvents
DMF	Dimethylformamide
DMSO	Dimethyl sulfoxide
DNA	Deoxyribonucleic Acid
ECM	Extracellular matrix
ES	Electrospinning
FDA	Food and Drug Administration
FE-SEM	Field emission scanning electron microscope
GVL	Gamma valerolactone
HFIP	Hexafluoro isopropanol
HP-β-CD	Hydroxypropyl-β-cyclodextrin
LCA	Life Cycle Assessment
LDH	Layered double hydroxide
MEK	Methyl ethyl ketone
MRSA	Methicillin-resistant *Staphylococcus aureus*
MRSE	Methicillin-resistant *Staphylococcus epidermidis*
NMMO	N-Methylmorpholine N-oxide
NPs	Nanoparticles
PAN	Polyacrylonitrile
PCL	Polycaprolactone
PDA	Polydopamine
PE	Polyethylene
PEG	Polyethylene glycol
PEO	Polyethylene oxide
PHA	Polyhydroxyalkanoate
PLA	Polylactic acid
PLGA	Poly(lactic-co-glycolic acid)
PLLA	Poly-l-lactic acid
PU	Polyurethane
PVA	Polyvinyl alcohol
PVDF	Polyvinylidene fluoride
PVDF-TrFE	Polyvinylidene fluoride trifluoro ethylene
SARS-CoV2	Severe acute respiratory syndrome coronavirus 2
SEM	Scanning electron microscopy
SF	Silk fibroin
TFA	Trifluoroacetic acid
TFE	Trifluoroethanol
UV	Ultraviolet
β-CD	β-cyclodextrin

## References

[1] Frieri M., Kumar K., Boutin A. (2017). Antibiotic resistance. J. Infect. Public Health.

[2] Jose A., Gizdavic-Nikolaidis M., Swift S. (2023). Antimicrobial Coatings: Reviewing Options for Healthcare Applications. Appl. Microbiol.

[3] Davies J., Davies D. (2010). Origins and Evolution of Antibiotic Resistance. Microbiol. Mol. Biol. Rev..

[4] Cassini A., Högberg L.D., Plachouras D., Quattrocchi A., Hoxha A., Simonsen G.S., Colomb-Cotinat M., Kretzschmar M.E., Devleesschauwer B., Cecchini M. (2019). Attributable deaths and disability-adjusted life-years caused by infections with antibiotic-resistant bacteria in the EU and the European Economic Area in 2015: A population-level modelling analysis. Lancet Infect. Dis..

[5] Singer A.C., Shaw H., Rhodes V., Hart A. (2016). Review of Antimicrobial Resistance in the Environment and Its Relevance to Environmental Regulators. Front. Microbiol.

[6] Larsson D.G.J., Flach C.-F. (2022). Antibiotic resistance in the environment. Nat. Rev. Microbiol..

[7] Kümmerer K., Dionysiou D.D., Olsson O., Fatta-Kassinos D. (2018). A path to clean water. Science.

[8] Li P., Wu Y., He Y., Zhang B., Huang Y., Yuan Q., Chen Y. (2020). Occurrence and fate of antibiotic residues and antibiotic resistance genes in a reservoir with ecological purification facilities for drinking water sources. Sci. Total Environ..

[9] Prestinaci F., Pezzotti P., Pantosti A. (2015). Antimicrobial resistance: A global multifaceted phenomenon. Pathog. Glob. Health.

[10] Aslam B., Wang W., Arshad M.I., Khurshid M., Muzammil S., Rasool M.H., Nisar M.A., Alvi R.F., Aslam M.A., Qamar M.U. (2018). Antibiotic resistance: A rundown of a global crisis. Infect. Drug Resist..

[11] Kraemer S.A., Ramachandran A., Perron G.G. (2019). Antibiotic Pollution in the Environment: From Microbial Ecology to Public Policy. Microorganisms.

[12] Ghafoor D., Khan Z., Khan A., Ualiyeva D., Zaman N. (2021). Excessive use of disinfectants against COVID-19 posing a potential threat to living beings. Curr. Res. Toxicol..

[13] Alaoui Mdarhri H., Benmessaoud R., Yacoubi H., Seffar L., Guennouni Assimi H., Hamam M., Boussettine R., Filali-Ansari N., Lahlou F.A., Diawara I. (2022). Alternatives Therapeutic Approaches to Conventional Antibiotics: Advantages, Limitations and Potential Application in Medicine. Antibiotics.

[14] Zarrintaj P., Seidi F., Youssefi Azarfam M., Khodadadi Yazdi M., Erfani A., Barani M., Chauhan N.P.S., Rabiee N., Kuang T., Kucinska-Lipka J. (2023). Biopolymer-based composites for tissue engineering applications: A basis for future opportunities. Compos. Part B Eng..

[15] Ambekar R.S., Kandasubramanian B. (2019). Advancements in nanofibers for wound dressing: A review. Eur. Polym. J..

[16] Rasouli R., Barhoum A., Bechelany M., Dufresne A. (2019). Nanofibers for Biomedical and Healthcare Applications. Macromol. Biosci..

[17] Hosseini E.S., Dervin S., Ganguly P., Dahiya R. (2021). Biodegradable Materials for Sustainable Health Monitoring Devices. ACS Appl. Bio Mater..

[18] Rai M.K., Deshmukh S.D., Ingle A.P., Gade A.K. (2012). Silver nanoparticles: The powerful nanoweapon against multidrug-resistant bacteria: Activity of silver nanoparticles against MDR bacteria. J. Appl. Microbiol..

[19] Zhang T., Gu J., Liu X., Wei D., Zhou H., Xiao H., Zhang Z., Yu H., Chen S. (2020). Bactericidal and antifouling electrospun PVA nanofibers modified with a quaternary ammonium salt and zwitterionic sulfopropylbetaine. Mater. Sci. Eng. C.

[20] Patra D., Ghosh S., Mukherjee S., Acharya Y., Mukherjee R., Haldar J. (2024). Antimicrobial nanocomposite coatings for rapid intervention against catheter-associated urinary tract infections. Nanoscale.

[21] Teixeira-Santos R., Lima M., Gomes L.C., Mergulhão F.J. (2021). Antimicrobial coatings based on chitosan to prevent implant-associated infections: A systematic review. iScience.

[22] Milazzo M., Gallone G., Marcello E., Mariniello M.D., Bruschini L., Roy I., Danti S. (2020). Biodegradable polymeric micro/nano-structures with intrinsic antifouling/antimicrobial properties: Relevance in damaged skin and other biomedical applications. J. Funct. Biomater..

[23] Maliszewska I., Czapka T. (2022). Electrospun Polymer Nanofibers with Antimicrobial Activity. Polymers.

[24] Salam M.A., Al-Amin M.Y., Salam M.T., Pawar J.S., Akhter N., Rabaan A.A., Alqumber M.A.A. (2023). Antimicrobial Resistance: A Growing Serious Threat for Global Public Health. Healthcare.

[25] Maillard J.-Y., Pascoe M. (2024). Disinfectants and antiseptics: Mechanisms of action and resistance. Nat. Rev. Microbiol..

[26] Mohsen S., Dickinson J.A., Somayaji R. (2020). Update on the adverse effects of antimicrobial therapies in community practice. Can. Fam. Physician.

[27] Spicer J.O., Roberts R.M., Hicks L.A. (2020). Perceptions of the Benefits and Risks of Antibiotics Among Adult Patients and Parents With High Antibiotic Utilization. Open Forum Infect. Dis..

[28] Abadi B., Goshtasbi N., Bolourian S., Tahsili J., Adeli-Sardou M., Forootanfar H. (2022). Electrospun hybrid nanofibers: Fabrication, characterization, and biomedical applications. Front. Bioeng. Biotechnol..

[29] White N.M., Barnett A.G., Hall L., Mitchell B.G., Farrington A., Halton K., Paterson D.L., Riley T.V., Gardner A., Page K. (2020). Cost-effectiveness of an Environmental Cleaning Bundle for Reducing Healthcare-associated Infections. Clin. Infect. Dis..

[30] De La Ossa J.G., Danti S., Esposito Salsano J., Azimi B., Tempesti V., Barbani N., Digiacomo M., Macchia M., Uddin M.J., Cristallini C. (2022). Electrospun Poly(3-Hydroxybutyrate-Co-3-Hydroxyvalerate)/Olive Leaf Extract Fiber Mesh as Prospective Bio-Based Scaffold for Wound Healing. Molecules.

[31] Azimi B., Ricci C., Macchi T., Günday C., Munafò S., Maleki H., Pratesi F., Tempesti V., Cristallini C., Bruschini L. (2023). A Straightforward Method to Produce Multi-Nanodrug Delivery Systems for Transdermal/Tympanic Patches Using Electrospinning and Electrospray. Polymers.

[32] Jian S., Zhu J., Jiang S., Chen S., Fang H., Song Y., Duan G., Zhang Y., Hou H. (2018). Nanofibers with diameter below one nanometer from electrospinning. RSC Adv..

[33] Leena M.M., Yoha K.S., Moses J.A., Anandharamakrishnan C., Knoerzer K., Muthukumarappan K. (2021). 3.37—Nanofibers in Food Applications. Innovative Food Processing Technologies.

[34] Cecchini B., Rovelli R., Zavagna L., Macchi T., Kaya E., Esin S., Bruschini L., Milazzo M., Batoni G., Danti S. (2023). Alginate-Based Patch for Middle Ear Delivery of Probiotics: A Preliminary Study Using Electrospray and Electrospinning. Appl. Sci..

[35] Azimi B., Labardi M., Sorayani Bafqi M.S., Macchi T., Ricci C., Carnicelli V., Scarpelli L., Hussain I., Matino F., Scaglione M. (2024). Remnant polarization and structural arrangement in P(VDF-TrFE) electrospun fiber meshes affect osteogenic differentiation of human mesenchymal stromal cells. Mater. Des..

[36] Kalaoglu-Altan O.I., Baskan H., Meireman T., Basnett P., Azimi B., Fusco A., Funel N., Donnarumma G., Lazzeri A., Roy I. (2021). Silver Nanoparticle-Coated Polyhydroxyalkanoate Based Electrospun Fibers for Wound Dressing Applications. Materials.

[37] Azimi B., Rasti A., Fusco A., Macchi T., Ricci C., Hosseinifard M.A., Guazzelli L., Donnarumma G., Bagherzadeh R., Latifi M. (2024). Bacterial Cellulose Electrospun Fiber Mesh Coated with Chitin Nanofibrils for Eardrum Repair. Tissue Eng. Part A.

[38] Asare E., Azimi B., Vasili E., Gregory D.A., Raut M., Taylor C.S., Linari S., Danti S., Roy I. (2025). Electrospun fibers of polyhydroxyalkanoate/bacterial cellulose blends and their role in nerve tissue engineering. Macromol. Mater. Eng..

[39] Maleki H., Azimi B., Ismaeilimoghadam S., Danti S. (2022). Poly (lactic acid)-Based Electrospun Fibrous Structures for Biomedical Applications. Appl. Sci..

[40] Dadol G.C., Kilic A., Tijing L.D., Lim K.J.A., Cabatingan L.K., Tan N.P.B., Stojanovska E., Polat Y. (2020). Solution blow spinning (SBS) and SBS-spun nanofibers: Materials, methods, and applications. Mater. Today Commun..

[41] Ji D., Xiao C., An S., Chen K., Gao Y., Zhou F., Zhang T. (2020). Completely green and sustainable preparation of PVDF hollow fiber membranes via melt-spinning and stretching method. J. Hazard. Mater..

[42] Chauvet M., Sauceau M., Fages J. (2017). Extrusion assisted by supercritical CO_2_: A review on its application to biopolymers. J. Supercrit. Fluids.

[43] Doshi J., Reneker D.H. (1995). Electrospinning process and applications of electrospun fibers. J. Electrost..

[44] Kulkarni D., Musale S., Panzade P., Paiva-Santos A.C., Sonwane P., Madibone M., Choundhe P., Giram P., Cavalu S. (2022). Surface Functionalization of Nanofibers: The Multifaceted Approach for Advanced Biomedical Applications. Nanomaterials.

[45] Khan J., Khan A., Khan M.Q., Khan H. (2024). Applications of co-axial electrospinning in the biomedical field. Next Mater..

[46] Avershina E., Shapovalova V., Shipulin G. (2021). Fighting Antibiotic Resistance in Hospital-Acquired Infections: Current State and Emerging Technologies in Disease Prevention, Diagnostics and Therapy. Front. Microbiol..

[47] Homaeigohar S., Boccaccini A.R. (2020). Antibacterial biohybrid nanofibers for wound dressings. Acta Biomater..

[48] Chakrapani G., Ramakrishna S., Zare M. (2023). Functionalization of electrospun nanofiber for biomedical application. J. Appl. Polym. Sci..

[49] Venmathi Maran B.A., Jeyachandran S., Kimura M. (2024). A Review on the Electrospinning of Polymer Nanofibers and Its Biomedical Applications. Compos. Sci..

[50] Zhang M., Song W., Tang Y., Xu X., Huang Y., Yu D. (2022). Polymer-Based Nanofiber–Nanoparticle Hybrids and Their Medical Applications. Polymers.

[51] Abdulhussain R., Adebisi A., Conway B.R., Asare-Addo K. (2023). Electrospun nanofibers: Exploring process parameters, polymer selection, and recent applications in pharmaceuticals and drug delivery. J. Drug Deliv. Sci. Technol..

[52] Nourpanah P., Rabiee M., Arbab S., Cascone M.G., Baldassare A., Lazzeri L. (2015). Application of response surface methodology to evaluate the effect of dry-spinning parameters on poly (ε-caprolactone) fiber properties. J. Appl. Polym. Sci..

[53] Azimi B., Nourpanah P., Rabiee M., Arbab S., Grazia Cascone M., Baldassare A., Lazzeri L. (2016). Application of the dry-spinning method to produce poly(ε-caprolactone) fibers containing bovine serum albumin laden gelatin nanoparticles. J. Appl. Polym. Sci..

[54] Lin S.-H., Ou S.-L., Hsu H.-M., Wu J.-Y. (2023). Preparation and Characteristics of Polyethylene Oxide/Curdlan Nanofiber Films by Electrospinning for Biomedical Applications. Materials.

[55] Türkoğlu G.C., Khomarloo N., Mohsenzadeh E., Gospodinova D.N., Neznakomova M., Salaün F. (2024). PVA-Based Electrospun Materials—A Promising Route to Designing Nanofiber Mats with Desired Morphological Shape—A Review. Int. J. Mol. Sci..

[56] Wilk S., Benko A. (2021). Advances in Fabricating the Electrospun Biopolymer-Based Biomaterials. J. Funct. Biomater..

[57] Ricci C., Azimi B., Panariello L., Antognoli B., Cecchini B., Rovelli R., Rustembek M., Cinelli P., Milazzo M., Danti S. (2023). Assessment of Electrospun Poly (ε-caprolactone) and Poly (lactic acid) Fiber Scaffolds to Generate 3D In Vitro Models of Colorectal Adenocarcinoma: A Preliminary Study. Int. J. Mol. Sci..

[58] Azimi B., Milazzo M., Danti S. (2021). Cellulose-Based Fibrous Materials From Bacteria to Repair Tympanic Membrane Perforations. Front. Bioeng. Biotechnol..

[59] Gregory D.A., Taylor C.S., Fricker A.T.R., Asare E., Tetali S.S.V., Haycock J.W., Roy I. (2022). Polyhydroxyalkanoates and their advances for biomedical applications. Trends Mol. Med..

[60] Tamilarasi G.P., Sabarees G., Manikandan K., Gouthaman S., Alagarsamy V., Solomon V.R. (2023). Advances in electrospun chitosan nanofiber biomaterials for biomedical applications. Mater. Adv..

[61] Peranidze K., Safronova T.V., Kildeeva N.R. (2023). Electrospun Nanomaterials Based on Cellulose and Its Derivatives for Cell Cultures: Recent Developments and Challenges. Polymers.

[62] Sanhueza C., Acevedo F., Rocha S., Villegas P., Seeger M., Navia R. (2019). Polyhydroxyalkanoates as biomaterial for electrospun scaffolds. Int. J. Biol. Macromol..

[63] Preethi G.U., Joseph M.M., Unnikrishnan B.S., Shiji R., Sreelekha T.T. (2015). Biomedical applications of natural polymer based nanofibrous scaffolds. Int. J. Med. Nano Res..

[64] BaoLin G., MA P.X. (2014). Synthetic biodegradable functional polymers for tissue engineering: A brief review. Sci. China Chem..

[65] Baranwal J., Barse B., Fais A., Delogu G.L., Kumar A. (2022). Biopolymer: A Sustainable Material for Food and Medical Applications. Polymers.

[66] Monia T. (2024). Sustainable natural biopolymers for biomedical applications. J. Thermoplast. Compos. Mater..

[67] Bagdadi A.V., Safari M., Dubey P., Basnett P., Sofokleous P., Humphrey E., Locke I., Edirisinghe M., Terracciano C., Boccaccini A.R. (2018). Poly (3-hydroxyoctanoate), a promising new material for cardiac tissue engineering. J. Tissue Eng. Regen. Med..

[68] Lizarraga-Valderrama L.R., Ronchi G., Nigmatullin R., Fregnan F., Basnett P., Paxinou A., Geuna S., Roy I. (2021). Preclinical study of peripheral nerve regeneration using nerve guidance conduits based on polyhydroxyalkanaotes. Bioeng. Transl. Med..

[69] Misra S.K., Valappil S.P., Roy I., Boccaccini A.R. (2006). Polyhydroxyalkanoate (PHA)/Inorganic Phase Composites for Tissue Engineering Applications. Biomacromolecules.

[70] Nigmatullin R., Thomas P., Lukasiewicz B., Puthussery H., Roy I. (2015). Polyhydroxyalkanoates, a family of natural polymers, and their applications in drug delivery. J. Chem. Technol. Biotechnol..

[71] Zhang Y., Zhang C., Wang Y. (2021). Recent progress in cellulose-based electrospun nanofibers as multifunctional materials. Nanoscale Adv..

[72] Bhat A.H., Khan I., Usmani M.A., Umapathi R., Al-Kindy S.M.Z. (2019). Cellulose an ageless renewable green nanomaterial for medical applications: An overview of ionic liquids in extraction, separation and dissolution of cellulose. Int. J. Biol. Macromol..

[73] Seddiqi H., Oliaei E., Honarkar H., Jin J., Geonzon L.C., Bacabac R.G., Klein-Nulend J. (2021). Cellulose and its derivatives: Towards biomedical applications. Cellulose.

[74] Norrrahim M.N.F., Nurazzi N.M., Jenol M.A., Farid M.A.A., Janudin N., Ujang F.A., Yasim-Anuar T.A.T., Syed Najmuddin S.U.F., Ilyas R.A. (2021). Emerging development of nanocellulose as an antimicrobial material: An overview. Mater. Adv..

[75] Phan D.-N., Khan M.Q., Nguyen V.-C., Vu-Manh H., Dao A.-T., Thanh Thao P., Nguyen N.-M., Le V.-T., Ullah A., Khatri M. (2021). Investigation of Mechanical, Chemical, and Antibacterial Properties of Electrospun Cellulose-Based Scaffolds Containing Orange Essential Oil and Silver Nanoparticles. Polymers.

[76] Thomas B., Raj M.C., Athira K.B., Rubiyah M.H., Joy J., Moores A., Drisko G.L., Sanchez C. (2018). Nanocellulose, a Versatile Green Platform: From Biosources to Materials and Their Applications. Chem. Rev..

[77] Kramar A., González-Benito F.J. (2022). Cellulose-Based Nanofibers Processing Techniques and Methods Based on Bottom-Up Approach—A Review. Polymers.

[78] Gregory D.A., Tripathi L., Fricker A.T.R., Asare E., Orlando I., Raghavendran V., Roy I. (2021). Bacterial cellulose: A smart biomaterial with diverse applications. Mater. Sci. Eng. R Rep..

[79] Abourehab M.A.S., Pramanik S., Abdelgawad M.A., Abualsoud B.M., Kadi A., Ansari M.J., Deepak A. (2022). Recent Advances of Chitosan Formulations in Biomedical Applications. Int. J. Mol. Sci..

[80] Santoro M., Shah S.R., Walker J.L., Mikos A.G. (2016). Poly (lactic acid) nanofibrous scaffolds for tissue engineering. Adv. Drug Deliv. Rev..

[81] Basnett P., Matharu R.K., Taylor C.S., Illangakoon U., Dawson J.I., Kanczler J.M., Behbehani M., Humphrey E., Majid Q., Lukasiewicz B. (2021). Harnessing Polyhydroxyalkanoates and Pressurized Gyration for Hard and Soft Tissue Engineering. ACS Appl. Mater. Interfaces.

[82] Ramezani Dana H., Ebrahimi F. (2023). Synthesis, properties, and applications of polylactic acid-based polymers. Polym. Eng. Sci..

[83] Dinjaski N., Fernández-Gutiérrez M., Selvam S., Parra-Ruiz F.J., Lehman S.M., San Román J., García E., García J.L., García A.J., Prieto M.A. (2014). PHACOS, a functionalized bacterial polyester with bactericidal activity against methicillin-resistant Staphylococcus aureus. Biomaterials.

[84] Do Val Siqueira L., Arias C.I.L.F., Maniglia B.C., Tadini C.C. (2021). Starch-based biodegradable plastics: Methods of production, challenges and future perspectives. Curr. Opin. Food Sci..

[85] Diaz-Baca J.A., Fatehi P. (2024). Production and characterization of starch-lignin based materials: A review. Biotechnol. Adv..

[86] Varanko A., Saha S., Chilkoti A. (2020). Recent trends in protein and peptide-based biomaterials for advanced drug delivery. Adv. Drug Deliv. Rev..

[87] Nie L., Hou R., Shavandi A. (2022). Editorial: Advances in protein-based biomaterials for tissue engineering. Front. Bioeng. Biotechnol..

[88] Zdraveva E., Gaurina Srček V., Kraljić K., Škevin D., Slivac I., Obranović M. (2023). Agro-industrial plant proteins in electrospun materials for biomedical application. Polymers.

[89] Marszalik K., Polak M., Knapczyk-Korczak J., Berniak K., Ibrahim M.N., Su Q., Li X., Ding B., Stachewicz U. (2025). Skin Regeneration and Wound Healing by Plant Protein-Based Electrospun Fiber Scaffolds and Patches for Tissue Engineering Applications. Macromol. Rapid Commun..

[90] Kalouta K., Stie M.B., Sun X., Foderà V., Vetri V. (2024). Eco-friendly electrospun nanofibers based on plant proteins as tunable and sustainable biomaterials. ACS Sustain. Chem. Eng..

[91] Qian W., Guo Y., Wang X., Qiu X., Ji X., Wang L., Li Y. (2022). Quantification and assessment of chemical footprint of VOCs in polyester fabric production. J. Clean. Prod..

[92] Avossa J., Herwig G., Toncelli C., Itel F., Rossi R.M. (2022). Electrospinning based on benign solvents: Current definitions, implications and strategies. Green Chem..

[93] Mosher C.Z., Brudnicki P.A.P., Gong Z., Childs H.R., Lee S.W., Antrobus R.M., Fang E.C., Schiros T.N., Lu H.H. (2021). Green electrospinning for biomaterials and biofabrication. Biofabrication.

[94] Thamer B.M., Al-Sabri A.E., Almansob A., El-Newehy M.H. (2022). Fabrication of Biohybrid Nanofibers by the Green Electrospinning Technique and Their Antibacterial Activity. ACS Omega.

[95] Badran R., Gharios R., Tehrani-Bagha A.R., Horzum N., Demir M.M., Muñoz-Espí R., Crespy D. (2019). 6. Water-based electrospinning. Green Electrospinning.

[96] Azimi B., Maleki H., Gigante V., Bagherzadeh R., Mezzetta A., Milazzo M., Guazzelli L., Cinelli P., Lazzeri A., Danti S. (2022). Cellulose-based fiber spinning processes using ionic liquids. Cellulose.

[97] Paiva A., Craveiro R., Aroso I., Martins M., Reis R.L., Duarte A.R.C. (2014). Natural Deep Eutectic Solvents—Solvents for the 21st Century. ACS Sustain. Chem. Eng..

[98] Nkuku C.A., LeSuer R.J. (2007). Electrochemistry in Deep Eutectic Solvents. J. Phys. Chem. B.

[99] Sousa A.M.M., Souza H.K.S., Uknalis J., Liu S.-C., Gonçalves M.P., Liu L. (2015). Improving agar electrospinnability with choline-based deep eutectic solvents. Int. J. Biol. Macromol..

[100] Vasili E., Azimi B., Raut M.P., Gregory D.A., Mele A., Liu B., Römhild K., Krieg M., Claeyssens F., Cinelli P. (2025). A Green Method for Bacterial Cellulose Electrospinning Using 1-Butyl-3-Methylimidazolium Acetate and γ-Valerolactone. Polymers.

[101] Li L., Jiang Z., Pan Q., Fang T. (2013). Producing polymer fibers by electrospinning in supercritical fluids. J. Chem..

[102] Hossain M.M., Islam T., Jalil M.A., Rakibuzzaman S.M., Surid S.M., Zabed M.R.I., Talukder A., Hossain S. (2024). Advancements of eco-friendly natural antimicrobial agents and their transformative role in sustainable textiles. SPE Polym..

[103] Berdimurodov E., Dagdag O., Berdimuradov K., Wan Nik W.M.N., Eliboev I., Ashirov M., Niyozkulov S., Demir M., Yodgorov C., Aliev N. (2023). Green Electrospun Nanofibers for Biomedicine and Biotechnology. Technologies.

[104] Arenbergerova M., Arenberger P., Bednar M., Kubat P., Mosinger J. (2012). Light-activated nanofibre textiles exert antibacterial effects in the setting of chronic wound healing. Exp. Dermatol..

[105] Spasova M., Paneva D., Manolova N., Radenkov P., Rashkov I. (2008). Electrospun Chitosan-Coated Fibers of Poly (L-lactide) and Poly (L-lactide)/Poly (ethylene glycol): Preparation and Characterization. Macromol. Biosci..

[106] Sun D., Babar Shahzad M., Li M., Wang G., Xu D. (2015). Antimicrobial materials with medical applications. Mater. Technol..

[107] Topcu B., Gultekinoglu M., Timur S.S., Eroglu I., Ulubayram K., Eroglu H. (2021). Current approaches and future prospects of nanofibers: A special focus on antimicrobial drug delivery. J. Drug Target..

[108] Aravind M., Amalanathan M., Aslam S., Noor A.E., Jini D., Majeed S., Velusamy P., Alothman A.A., Alshgari R.A., Saleh Mushab M.S. (2023). Hydrothermally synthesized Ag-TiO2 nanofibers (NFs) for photocatalytic dye degradation and antibacterial activity. Chemosphere.

[109] Wei Z., Liu E., Li H., Wei Z., Lv Z. (2021). Release characteristics of different diameter ultrafine fibers as antibacterial materials. J. Innov. Opt. Health Sci..

[110] Nitti P., Gallo N., Natta L., Scalera F., Palazzo B., Sannino A., Gervaso F. (2018). Influence of Nanofiber Orientation on Morphological and Mechanical Properties of Electrospun Chitosan Mats. J. Healthc. Eng..

[111] Mercante L., Teodoro K., Dos Santos D., Dos Santos F., Ballesteros C., Ju T., Williams G., Correa D. (2023). Recent Progress in Stimuli-Responsive Antimicrobial Electrospun Nanofibers. Polymers.

[112] Dash M., Chiellini F., Ottenbrite R.M., Chiellini E. (2011). Chitosan—A versatile semi-synthetic polymer in biomedical applications. Prog. Polym. Sci..

[113] Villarreal-Gómez L.J., Pérez-González G.L., Bogdanchikova N., Pestryakov A., Nimaev V., Soloveva A., Cornejo-Bravo J.M., Toledaño-Magaña Y. (2021). Antimicrobial Effect of Electrospun Nanofibers Loaded with Silver Nanoparticles: Influence of Ag Incorporation Method. J. Nanomater..

[114] Sabarees G., Velmurugan V., Tamilarasi G.P., Alagarsamy V., Raja Solomon V. (2022). Recent Advances in Silver Nanoparticles Containing Nanofibers for Chronic Wound Management. Polymers.

[115] Yılmaz G.E., Göktürk I., Ovezova M., Yılmaz F., Kılıç S., Denizli A. (2023). Antimicrobial Nanomaterials: A Review. Hygiene.

[116] Haider M.K., Ullah A., Sarwar M.N., Yamaguchi T., Wang Q., Ullah S., Park S., Kim I.S. (2021). Fabricating Antibacterial and Antioxidant Electrospun Hydrophilic Polyacrylonitrile Nanofibers Loaded with AgNPs by Lignin-Induced In-Situ Method. Polymers.

[117] Kielholz T., Walther M., Jung N., Windbergs M. (2022). Electrospun fibers loaded with antimicrobial peptides for treatment of wound infections. Eur. J. Pharm. Biopharm..

[118] Xuan J., Feng W., Wang J., Wang R., Zhang B., Bo L., Chen Z.-S., Yang H., Sun L. (2023). Antimicrobial peptides for combating drug-resistant bacterial infections. Drug Resist. Updat..

[119] Hamdan N., Yamin A., Hamid S.A., Khodir W.K.W.A., Guarino V. (2021). Functionalized Antimicrobial Nanofibers: Design Criteria and Recent Advances. J. Funct. Biomater..

[120] Li W., Thian E.S., Wang M., Wang Z., Ren L. (2021). Surface Design for Antibacterial Materials: From Fundamentals to Advanced Strategies. Adv. Sci..

[121] Jiao Y., Niu L., Ma S., Li J., Tay F.R., Chen J. (2017). Quaternary ammonium-based biomedical materials: State-of-the-art, toxicological aspects and antimicrobial resistance. Prog. Polym. Sci..

[122] Ojah N., Borah R., Ahmed G.A., Mandal M., Choudhury A.J. (2020). Surface modification of electrospun silk/AMOX/PVA nanofibers by dielectric barrier discharge plasma: Physiochemical properties, drug delivery and in-vitro biocompatibility. Prog. Biomater..

[123] Chong W.J., Shen S., Li Y., Trinchi A., Pejak Simunec D., Kyratzis I., Sola A., Wen C. (2023). Biodegradable PLA-ZnO nanocomposite biomaterials with antibacterial properties, tissue engineering viability, and enhanced biocompatibility. Smart Mater. Manuf..

[124] Kai D., Liow S.S., Loh X.J. (2014). Biodegradable polymers for electrospinning: Towards biomedical applications. Mater. Sci. Eng. C.

[125] Azimi B., Sorayani Bafqi M.S., Fusco A., Ricci C., Gallone G., Bagherzadeh R., Donnarumma G., Uddin M.J., Latifi M., Lazzeri A. (2020). Electrospun ZnO/Poly (Vinylidene Fluoride-Trifluoroethylene) Scaffolds for Lung Tissue Engineering. Tissue Eng. Part A.

[126] Maximiano M.R., Rios T.B., Campos M.L., Prado G.S., Dias S.C., Franco O.L. (2022). Nanoparticles in association with antimicrobial peptides (NanoAMPs) as a promising combination for agriculture development. Front. Mol. Biosci..

[127] Taiswa A., Andriolo J.M., Hailer M.K., Skinner J.L. (2024). Quorum Quenching Nanofibers for Anti-Biofouling Applications. Coatings.

[128] Khan R.S., Rather A.H., Wani T.U., Rafiq M., Sheikh F.A. (2023). Efficiency of Prevention of Biofilm Formation by Modified Polyurethane Nanofibers in Different Bacterial Strains. J. Phys. Conf. Ser..

[129] Sethuram L., Thomas J. (2023). Therapeutic applications of electrospun nanofibers impregnated with various biological macromolecules for effective wound healing strategy—A review. Biomed. Pharmacother..

[130] Azimi B., Maleki H., Zavagna L., De La Ossa J.G., Linari S., Lazzeri A., Danti S. (2020). Bio-Based Electrospun Fibers for Wound Healing. J. Funct. Biomater..

[131] Kadirvelu L., Sivaramalingam S.S., Jothivel D., Chithiraiselvan D.D., Karaiyagowder Govindarajan D., Kandaswamy K. (2024). A review on antimicrobial strategies in mitigating biofilm-associated infections on medical implants. Curr. Res. Microb. Sci..

[132] Li J., Cheung W.-H., Chow S.K., Ip M., Leung S.Y.S., Wong R.M.Y. (2022). Current therapeutic interventions combating biofilm-related infections in orthopaedics: A systematic review of in vivo animal studies. Bone Jt. Res..

[133] George N., Chakraborty S., Mary N.L., Suguna L. (2024). Incorporating silver nanoparticles into electrospun nanofibers of casein/polyvinyl alcohol to develop scaffolds for tissue engineering. Int. J. Biol. Macromol..

[134] Muñoz-Bonilla A., Echeverria C., Sonseca Á., Arrieta M.P., Fernández-García M. (2019). Bio-Based Polymers with Antimicrobial Properties towards Sustainable Development. Materials.

[135] Teixeira M.A., Amorim M.T.P., Felgueiras H.P. (2019). Poly (Vinyl Alcohol)-Based Nanofibrous Electrospun Scaffolds for Tissue Engineering Applications. Polymers.

[136] Kim H.-K., Jang S.-J., Cho Y.-S., Park H.-H. (2022). Fabrication of Nanostructured Polycaprolactone (PCL) Film Using a Thermal Imprinting Technique and Assessment of Antibacterial Function for Its Application. Polymers.

[137] Latiffah E., Sawitri A., Agung B.H., Hapidin D.A., Edikresnha D., Elfahmi E., Khairurrijal K. (2024). Antibacterial activity of electrospun nanofibers polyvinylpyrrolidone/cellulose acetate matrix loaded by Ageratum conyzoides L. weed. Case Stud. Chem. Environ. Eng..

[138] Caracciolo P.C., Abraham G.A., Battaglia E.S., Bongiovanni Abel S. (2023). Recent Progress and Trends in the Development of Electrospun and 3D Printed Polymeric-Based Materials to Overcome Antimicrobial Resistance (AMR). Pharmaceutics.

[139] Shepa I., Mudra E., Pavlinak D., Antal V., Bednarcik J., Mikovic O., Kovalcikova A., Dusza J. (2020). Surface plasma treatment of the electrospun TiO2/PVP composite fibers in different atmospheres. Appl. Surf. Sci..

[140] Hsu Y.-H., Yu Y.-H., Chou Y.-C., Lu C.-J., Lin Y.-T., Ueng S.W.-N., Liu S.-J. (2023). Sustained Release of Antifungal and Antibacterial Agents from Novel Hybrid Degradable Nanofibers for the Treatment of Polymicrobial Osteomyelitis. Int. J. Mol. Sci..

[141] Van-Pham D.-T., Thi Bich Quyen T., Van Toan P., Nguyen C.-N., Ho M.H., Van Hong Thien D. (2020). Temperature effects on electrospun chitosan nanofibers. Green Process. Synth..

[142] Ardean C., Davidescu C.M., Nemeş N.S., Negrea A., Ciopec M., Duteanu N., Negrea P., Duda-Seiman D., Musta V. (2021). Factors Influencing the Antibacterial Activity of Chitosan and Chitosan Modified by Functionalization. Int. J. Mol. Sci..

[143] Sayin S., Tufani A., Emanet M., Genchi G.G., Sen O., Shemshad S., Ozdemir E., Ciofani G., Ozaydin Ince G. (2019). Electrospun Nanofibers With pH-Responsive Coatings for Control of Release Kinetics. Front. Bioeng. Biotechnol..

[144] Kamsani N.H., Haris M.S., Pandey M., Taher M., Rullah K. (2021). Biomedical application of responsive ‘smart’ electrospun nanofibers in drug delivery system: A minireview. Arab. J. Chem..

[145] Makabenta J.M.V., Nabawy A., Li C.-H., Schmidt-Malan S., Patel R., Rotello V.M. (2021). Nanomaterial-based therapeutics for antibiotic-resistant bacterial infections. Nat. Rev. Microbiol..

[146] Pang Q., Jiang Z., Wu K., Hou R., Zhu Y. (2023). Nanomaterials-Based Wound Dressing for Advanced Management of Infected Wound. Antibiotics.

[147] Aabed K., Mohammed A.E. (2021). Synergistic and Antagonistic Effects of Biogenic Silver Nanoparticles in Combination With Antibiotics Against Some Pathogenic Microbes. Front. Bioeng. Biotechnol..

[148] Hao Z., Wang M., Cheng L., Si M., Feng Z., Feng Z. (2024). Synergistic antibacterial mechanism of silver-copper bimetallic nanoparticles. Front. Bioeng. Biotechnol..

[149] Sharma D., Gautam S., Singh S., Srivastava N., Khan A.M., Bisht D. (2025). Unveiling the nanoworld of antimicrobial resistance: Integrating nature and nanotechnology. Front. Microbiol..

[150] Neščáková Z., Kaňková H., Galusková D., Galusek D., Boccaccini A.R., Liverani L. (2021). Polymer (PCL) fibers with Zn-doped mesoporous bioactive glass nanoparticles for tissue regeneration. Int. J. Appl. Glass Sci..

[151] Zhou J., Li X., Zhang Z., Hou T., Xu J., Wang Y., Ye H., Yang B. (2024). Bio-based and bio-degradable nanofiber materials: A sustainable platform for energy, environmental, and biomedical applications. Chem. Eng. J..

[152] Gavande V., Nagappan S., Seo B., Lee W.-K. (2024). A systematic review on green and natural polymeric nanofibers for biomedical applications. Int. J. Biol. Macromol..

[153] Kong B., Liu R., Guo J., Lu L., Zhou Q., Zhao Y. (2023). Tailoring micro/nano-fibers for biomedical applications. Bioact. Mater..

[154] Berglund L., Breedveld L., Oksman K. (2020). Toward eco-efficient production of natural nanofibers from industrial residue: Eco-design and quality assessment. J. Clean. Prod..

[155] Yates M.R., Barlow C.Y. (2013). Life cycle assessments of biodegradable, commercial biopolymers—A critical review. Resour. Conserv. Recycl..

[156] Amarakoon M., Alenezi H., Homer-Vanniasinkam S., Edirisinghe M. (2022). Environmental impact of polymer fiber manufacture. Macromol. Mater. Eng..

[157] Unger S.R., Hottle T.A., Hobbs S.R., Thiel C.L., Campion N., Bilec M.M., Landis A.E. (2017). Do single-use medical devices containing biopolymers reduce the environmental impacts of surgical procedures compared with their plastic equivalents?. J. Health Serv. Res. Policy.

[158] Moshkbid E., Cree D.E., Bradford L., Zhang W. (2024). Biodegradable Alternatives to Plastic in Medical Equipment: Current State, Challenges, and the Future. J. Compos. Sci..

[159] Samir A., Ashour F.H., Hakim A.A.A., Bassyouni M. (2022). Recent advances in biodegradable polymers for sustainable applications. npj Mater. Degrad..

[160] Iurilli M., Porrelli D., Turco G., Lagatolla C., Camurri Piloni A., Medagli B., Nicolin V., Papa G. (2025). Electrospun Collagen-Coated Nanofiber Membranes Functionalized with Silver Nanoparticles for Advanced Wound Healing Applications. Membranes.

[161] Zhang Y., Lu L., Chen Y., Wang J., Chen Y., Mao C., Yang M. (2019). Polydopamine modification of silk fibroin membranes significantly promotes their wound healing effect. Biomater. Sci..

[162] Parkale R., Pulugu P., Kumar P. (2021). Nanomaterials decoration on commercial cotton bandages for pain and infection management. arXiv.

[163] Parkale R., Pulugu P., Kumar P. (2023). Developing easy-to-use, cost-effective wound dressing material by coating commercial cotton bandages with nanomaterials. Int. J. Mater. Res..

[164] Bagheri Azizabad Z., Haghbin Nazarpak M., Nayeb Habib F. (2023). Modification of cotton gauze by electrospinning of gelatin and honey biopolymer solution. J. Text. Inst..

[165] Bulman S.E., Goswami P., Tronci G., Russell S.J., Carr C. (2015). Investigation into the potential use of poly (vinyl alcohol)/methylglyoxal fibres as antibacterial wound dressing components. J. Biomater. Appl..

[166] Parın F.N., Yeşilyurt A., Parın U. (2024). PVA/Whey Protein Nanofiber-Coated Pp Melt Blown Integrated With Pickering Emulsion of Citral Stabilized for Potential Medical Applications. Int. J. Innov. Eng. Appl..

[167] Guha Ray P., Pal P., Srivas P.K., Basak P., Roy S., Dhara S. (2018). Surface Modification of Eggshell Membrane with Electrospun Chitosan/Polycaprolactone Nanofibers for Enhanced Dermal Wound Healing. ACS Appl. Bio Mater..

[168] Mouro C., Dunne C.P., Gouveia I.C. (2020). Designing New Antibacterial Wound Dressings: Development of a Dual Layer Cotton Material Coated with Poly (Vinyl Alcohol) Chitosan Nanofibers Incorporating Agrimonia eupatoria L. Extract. Molecules.

[169] Nawalakhe R., Shi Q., Vitchuli N., Noar J., Caldwell J.M., Breidt F., Bourham M.A., Zhang X., McCord M.G. (2013). Novel atmospheric plasma enhanced chitosan nanofiber/gauze composite wound dressings. J. Appl. Polym. Sci..

[170] Patil N.A., Gore P.M., Jaya Prakash N., Govindaraj P., Yadav R., Verma V., Shanmugarajan D., Patil S., Kore A., Kandasubramanian B. (2021). Needleless electrospun phytochemicals encapsulated nanofibre based 3-ply biodegradable mask for combating COVID-19 pandemic. Chem. Eng. J..

[171] Monavari M., Zohoori S., Davodiroknabadi A. (2022). Anti-inflammatory and bactericidal effect of keratin/harmaline/ginkgo biloba electrospun nano fibres as band aid. Micro Nano Lett..

[172] Hassan M.M., Heins K., Zheng H. (2024). Wound Dressing Based on Silver Nanoparticle Embedded Wool Keratin Electrospun Nanofibers Deposited on Cotton Fabric: Preparation, Characterization, Antimicrobial Activity, and Cytocompatibility. ACS Appl. Bio Mater..

[173] Alishahi M., Aboelkheir M., Chowdhury R., Altier C., Shen H., Uyar T. (2024). Functionalization of cotton nonwoven with cyclodextrin/lawsone inclusion complex nanofibrous coating for antibacterial wound dressing. Int. J. Pharm..

[174] Jahanmard F., Croes M., Castilho M., Majed A., Steenbergen M.J., Lietaert K., Vogely H.C., Van Der Wal B.C.H., Stapels D.A.C., Malda J. (2020). Bactericidal coating to prevent early and delayed implant-related infections. J. Control. Release.

[175] Suchý T., Vištejnová L., Šupová M., Klein P., Bartoš M., Kolinko Y., Blassová T., Tonar Z., Pokorný M., Sucharda Z. (2021). Vancomycin-Loaded Collagen/Hydroxyapatite Layers Electrospun on 3D Printed Titanium Implants Prevent Bone Destruction Associated with S. epidermidis Infection and Enhance Osseointegration. Biomedicines.

[176] Li Z., Hu W., Zhao Y., Ren L., Yuan X. (2018). Integrated antibacterial and antifouling surfaces via cross-linking chitosan- g -eugenol/zwitterionic copolymer on electrospun membranes. Colloids Surf. B Biointerfaces.

[177] Ashbaugh A.G., Jiang X., Zheng J., Tsai A.S., Kim W.-S., Thompson J.M., Miller R.J., Shahbazian J.H., Wang Y., Dillen C.A. (2016). Polymeric nanofiber coating with tunable combinatorial antibiotic delivery prevents biofilm-associated infection in vivo. Proc. Natl. Acad. Sci. USA.

[178] Tuson H.H., Weibel D.B. (2013). Bacteria–surface interactions. Soft Matter.

[179] Sukhorukova I.V., Sheveyko A.N., Kiryukhantsev-Korneev P.V., Zhitnyak I.Y., Gloushankova N.A., Denisenko E.A., Filippovich S.Y., Ignatov S.G., Shtansky D.V. (2015). Toward bioactive yet antibacterial surfaces. Colloids Surf. B Biointerfaces.

[180] Permyakova E.S., Manakhov A.M., Kiryukhantsev-Korneev P.V., Sheveyko A.N., Gudz K.Y., Kovalskii A.M., Polčak J., Zhitnyak I.Y., Gloushankova N.A., Dyatlov I.A. (2021). Different concepts for creating antibacterial yet biocompatible surfaces: Adding bactericidal element, grafting therapeutic agent through COOH plasma polymer and their combination. Appl. Surf. Sci..

[181] Duan S., Wu R., Xiong Y.-H., Ren H.-M., Lei C., Zhao Y.-Q., Zhang X.-Y., Xu F.-J. (2022). Multifunctional antimicrobial materials: From rational design to biomedical applications. Prog. Mater. Sci..

[182] Zou Y., Zhang Y., Yu Q., Chen H. (2021). Dual-function antibacterial surfaces to resist and kill bacteria: Painting a picture with two brushes simultaneously. J. Mater. Sci. Technol..

[183] Raphel J., Holodniy M., Goodman S.B., Heilshorn S.C. (2016). Multifunctional coatings to simultaneously promote osseointegration and prevent infection of orthopaedic implants. Biomaterials.

[184] Goodman S.B., Yao Z., Keeney M., Yang F. (2013). The future of biologic coatings for orthopaedic implants. Biomaterials.

[185] Ma Y., Zhang Z., Nitin N., Sun G. (2020). Integration of photo-induced biocidal and hydrophilic antifouling functions on nanofibrous membranes with demonstrated reduction of biofilm formation. J. Colloid Interface Sci..

[186] Al-Enizi A.M., Zagho M.M., Elzatahry A.A. (2018). Polymer-Based Electrospun Nanofibers for Biomedical Applications. Nanomaterials.

[187] Li L., Wang L., Xu Y., Lv L. (2012). Preparation of gentamicin-loaded electrospun coating on titanium implants and a study of their properties in vitro. Arch. Orthop. Trauma Surg..

[188] Ansari M., Shahlaei M., Hosseinzadeh S., Moradi S. (2024). Recent advances in nanostructured delivery systems for vancomycin. Nanomedicine.

[189] Bakhsheshi-Rad H.R., Akbari M., Ismail A.F., Aziz M., Hadisi Z., Pagan E., Daroonparvar M., Chen X. (2019). Coating biodegradable magnesium alloys with electrospun poly-L-lactic acid-åkermanite-doxycycline nanofibers for enhanced biocompatibility, antibacterial activity, and corrosion resistance. Surf. Coat. Technol..

[190] Kiran A.S.K., Sampath Kumar T.S., Perumal G., Sanghavi R., Doble M., Ramakrishna S. (2018). Dual nanofibrous bioactive coating and antimicrobial surface treatment for infection resistant titanium implants. Prog. Org. Coat..

[191] Jatoi A.W., Kim I.S., Ni Q.Q. (2019). A comparative study on synthesis of AgNPs on cellulose nanofibers by thermal treatment and DMF for antibacterial activities. Mater. Sci. Eng. C.

[192] Al-Khateeb A., Al-Hassani E.S., Jabur A.R. (2023). Metallic Implant Surface Activation through Electrospinning Coating of Nanocomposite Fiber for Bone Regeneration. Int. J. Biomater..

[193] Pebdeni A.B., Sadri M., Pebdeni S.B. (2016). Synthesis of chitosan/PEO/silica nanofiber coating for controlled release of cefepime from implants. RSC Adv..

[194] Serpelloni S., Williams M.E., Caserta S., Sharma S., Rahimi M., Taraballi F. (2024). Electrospun Chitosan-Based Nanofibrous Coating for the Local and Sustained Release of Vancomycin. ACS Omega.

[195] Kharat Z., Sadri M., Kabiri M. (2021). Herbal Extract Loaded Chitosan/PEO Nanocomposites as Antibacterial Coatings of Orthopaedic Implants. Fibers Polym..

[196] Boschetto F., Ngoc Doan H., Phong Vo P., Zanocco M., Zhu W., Sakai W., Adachi T., Ohgitani E., Tsutsumi N., Mazda O. (2020). Antibacterial and Osteoconductive Effects of Chitosan/Polyethylene Oxide (PEO)/Bioactive Glass Nanofibers for Orthopedic Applications. Appl. Sci..

[197] Maver T., Mastnak T., Mihelič M., Maver U., Finšgar M. (2021). Clindamycin-Based 3D-Printed and Electrospun Coatings for Treatment of Implant-Related Infections. Materials.

[198] Ansari M.A., Albetran H.M., Alheshibri M.H., Timoumi A., Algarou N.A., Akhtar S., Slimani Y., Almessiere M.A., Alahmari F.S., Baykal A. (2020). Synthesis of Electrospun TiO2 Nanofibers and Characterization of Their Antibacterial and Antibiofilm Potential against Gram-Positive and Gram-Negative Bacteria. Antibiotics.

[199] Celebioglu A., Topuz F., Yildiz Z.I., Uyar T. (2019). One-step green synthesis of antibacterial silver nanoparticles embedded in electrospun cyclodextrin nanofibers. Carbohydr. Polym..

[200] Somogyi Škoc M., Meštrović E., Mouthuy P.-A., Rezić I. (2024). Synthesis, Characterization and Application of Advanced Antimicrobial Electrospun Polymers. Polymers.

[201] El-kaliuoby M.I., Khalil A.M., El-Khatib A.M., Shehata N. (2021). Antibacterial Synergism of Electrospun Nanofiber Mats Functioned with Silver Nanoparticles and Pulsed Electromagnetic Waves. Polymers.

[202] Mathur A., Kharbanda O.P., Koul V., Dinda A.K., Anwar M.F., Singh S. (2022). Fabrication and evaluation of antimicrobial biomimetic nanofiber coating for improved dental implant bioseal: An in vitro study. J. Periodontol..

[203] Liu F., Wang X., Chen T., Zhang N., Wei Q., Tian J., Wang Y., Ma C., Lu Y. (2020). Hydroxyapatite/silver electrospun fibers for anti-infection and osteoinduction. J. Adv. Res..

[204] Shahi R.G., Albuquerque M.T.P., Münchow E.A., Blanchard S.B., Gregory R.L., Bottino M.C. (2017). Novel bioactive tetracycline-containing electrospun polymer fibers as a potential antibacterial dental implant coating. Odontology.

[205] Rieger K., Porter M., Schiffman J. (2016). Polyelectrolyte-Functionalized Nanofiber Mats Control the Collection and Inactivation of Escherichia coli. Materials.

[206] Wang L., Zhang L., Yan J., Yin Z., Tang C., Guo Y., Li D., Wei B., Xu Y., Gu Q. (2014). Electrospun vancomycin-loaded coating on titanium implants for the prevention of implant-associated infections. Int. J. Nanomed..

[207] Favrin F.L., Zavagna L., Sestini M., Esin S., Azimi B., Labardi M., Milazzo M., Gallone G., Batoni G., Danti S. (2024). Antifouling Properties of Electrospun Polymeric Coatings Induced by Controlled Surface Morphology. Energy Environ. Mater..

[208] Monfared M.M., Aghdam R.M., Pishbin F., Rahmani M. (2024). Enhancement of PEO-coated ZK60 Mg alloy: Curcumin-enriched mesoporous silica and PLA/bioglass for antibacterial properties, bioactivity and biocorrosion resistance. Surf. Coat. Technol..

[209] Angelopoulos N., Staines J., Chamberlin M., Bates S., McGain F. (2025). A narrative review of personal protective equipment gowns: Lessons from COVID-19. Br. J. Anaesth..

[210] Rossin A.R.S., Spessato L., Cardoso F.D.S.L., Caetano J., Caetano W., Radovanovic E., Dragunski D.C. (2024). Electrospinning in personal protective equipment for healthcare work. Polym. Bull..

[211] Khan M.Q., Kharaghani D., Nishat N., Shahzad A., Hussain T., Khatri Z., Zhu C., Kim I.S. (2019). Preparation and characterizations of multifunctional PVA/ZnO nanofibers composite membranes for surgical gown application. J. Mater. Res. Technol..

[212] Salam A., Hassan T., Jabri T., Riaz S., Khan A., Iqbal K.M., Khan S.U., Wasim M., Shah M.R., Khan M.Q. (2021). Electrospun Nanofiber-Based Viroblock/ZnO/PAN Hybrid Antiviral Nanocomposite for Personal Protective Applications. Nanomaterials.

[213] Alshabanah L.A., Hagar M., Al-Mutabagani L.A., Abozaid G.M., Abdallah S.M., Ahmed H., Hassanin A.H., Shehata N. (2021). Biodegradable Nanofibrous Membranes for Medical and Personal Protection Applications: Manufacturing, Anti-COVID-19 and Anti-Multidrug Resistant Bacteria Evaluation. Materials.

[214] Fearon K.C.H., Ljungqvist O., Von Meyenfeldt M., Revhaug A., Dejong C.H.C., Lassen K., Nygren J., Hausel J., Soop M., Andersen J. (2005). Enhanced recovery after surgery: A consensus review of clinical care for patients undergoing colonic resection. Clin. Nutr..

[215] Khan S.A., Rahman Z.U., Cai Z., Jiang O., Xu G. (2024). Drug-eluting ureteral stents: An overview. J. Drug Deliv. Sci. Technol..

[216] Wang M., Tang T. (2019). Surface treatment strategies to combat implant-related infection from the beginning. J. Orthop. Transl..

[217] Ilie V.G., Ilie V.I. (2018). Ureteric Stent Use—Part of the Solution and Part of the Problem. Curr. Urol..

[218] Madalosso H.B., Machado R., Hotza D., Marangoni C. (2021). Membrane Surface Modification by Electrospinning, Coating, and Plasma for Membrane Distillation Applications: A State-of-the-Art Review. Adv. Eng. Mater..

[219] Korniienko V., Husak Y., Radwan-Pragłowska J., Holubnycha V., Samokhin Y., Yanovska A., Varava J., Diedkova K., Janus Ł., Pogorielov M. (2022). Impact of Electrospinning Parameters and Post-Treatment Method on Antibacterial and Antibiofilm Activity of Chitosan Nanofibers. Molecules.

[220] Hadi Salih H., Kadim Abid AlSahib N., Ismael Jawad H., Shokrolahi F. (2025). Engineering an Electrospun Composite Ureteral Stent Based on PLGA with Antibacterial Properties and Tailored Mechanical Strength. ACS Omega.

[221] Zhang Y., Wang L., Wang Y., Li L., Zhou J., Dou D., Wu Z., Yu L., Fan Y. (2023). Degradable Antimicrobial Ureteral Stent Construction with Silver@graphdiyne Nanocomposite. Adv. Healthc. Mater..

[222] Horzum N., Boyacı E., Eroğlu A.E., Shahwan T., Demir M.M. (2010). Sorption Efficiency of Chitosan Nanofibers toward Metal Ions at Low Concentrations. Biomacromolecules.

[223] Sheikh F.A., Ju H.W., Lee J.M., Moon B.M., Park H.J., Lee O.J., Kim J.-H., Kim D.-K., Park C.H. (2015). 3D electrospun silk fibroin nanofibers for fabrication of artificial skin. Nanomed. Nanotechnol. Biol. Med..

[224] Grimmelsmann N., Homburg S.V., Ehrmann A. (2017). Needleless Electrospinning of Pure and Blended Chitosan. IOP Conf. Ser. Mater. Sci. Eng..

[225] Antaby E., Klinkhammer K., Sabantina L. (2021). Electrospinning of Chitosan for Antibacterial Applications—Current Trends. Appl. Sci..

[226] Balakrishnan P., Geethamma V.G., Sreekala M.S., Thomas S. (2018). Polymeric biomaterials: State-of-the-art and new challenges. Fundamental Biomaterials: Polymers.

[227] Miguel S.P., Sequeira R.S., Moreira A.F., Cabral C.S.D., Mendonça A.G., Ferreira P., Correia I.J. (2019). An overview of electrospun membranes loaded with bioactive molecules for improving the wound healing process. Eur. J. Pharm. Biopharm..

[228] Adamu B.F., Gao J., Jhatial A.K., Kumelachew D.M. (2021). A review of medicinal plant-based bioactive electrospun nano fibrous wound dressings. Mater. Des..

[229] Suwantong O., Opanasopit P., Ruktanonchai U., Supaphol P. (2007). Electrospun cellulose acetate fiber mats containing curcumin and release characteristic of the herbal substance. Polymer.

[230] Chen K., Li Y., Li Y., Tan Y., Liu Y., Pan W., Tan G. (2023). Stimuli-responsive electrospun nanofibers for drug delivery, cancer therapy, wound dressing, and tissue engineering. J. Nanobiotechnol..

[231] Wu Z., Chan B., Low J., Chu J.J.H., Hey H.W.D., Tay A. (2022). Microbial resistance to nanotechnologies: An important but understudied consideration using antimicrobial nanotechnologies in orthopaedic implants. Bioact. Mater..

[232] Muteeb G., Rehman M.T., Shahwan M., Aatif M. (2023). Origin of Antibiotics and Antibiotic Resistance, and Their Impacts on Drug Development: A Narrative Review. Pharmaceuticals.

[233] Palani N., Vijayakumar P., Monisha P., Ayyadurai S., Rajadesingu S. (2024). Electrospun nanofibers synthesized from polymers incorporated with bioactive compounds for wound healing. J. Nanobiotechnol..

[234] Maduna L., Patnaik A. (2024). Challenges Associated with the Production of Nanofibers. Processes.

[235] Alvarez M.M., Aizenberg J., Analoui M., Andrews A.M., Bisker G., Boyden E.S., Kamm R.D., Karp J.M., Mooney D.J., Oklu R. (2017). Emerging Trends in Micro- and Nanoscale Technologies in Medicine: From Basic Discoveries to Translation. ACS Nano.

[236] Molla G., Bitew M. (2024). Revolutionizing Personalized Medicine: Synergy with Multi-Omics Data Generation, Main Hurdles, and Future Perspectives. Biomedicines.

[237] Ma X., Tian Y., Yang R., Wang H., Allahou L.W., Chang J., Williams G., Knowles J.C., Poma A. (2024). Nanotechnology in healthcare, and its safety and environmental risks. J. Nanobiotechnol..

[238] Chen Q.Z., Thompson I.D., Boccaccini A.R. (2006). 45S5 Bioglass^®^-derived glass–ceramic scaffolds for bone tissue engineering. Biomaterials.

